# Thwarted belongingness and empathy's relation with organizational culture change

**DOI:** 10.3389/fpsyg.2024.1287769

**Published:** 2024-04-04

**Authors:** Éloïse de Grandpré, Cindy Suurd Ralph, Emily Hiller

**Affiliations:** Department of Military Psychology and Leadership, Royal Military College of Canada, Kingston, ON, Canada

**Keywords:** organizational culture change, empathy, military academies, thwarted belongingness, inclusion

## Abstract

**Introduction:**

In response to several high-profile cases of senior leaders in the Canadian Armed Forces (CAF) being accused of various forms of sexual and professional misconduct, the organization has committed to culture change. Drawing on the group engagement model and empirical evidence, we propose that CAF members' experience of thwarted belongingness reduces their capacity to show empathy, which in turn affects their support for culture change.

**Method:**

Participants were 139 Naval and Officer Cadets from the Royal Military College of Canada who were predominantly male (61%), between 18 and 21 years old (80%), and not members of a visible minority group (68%). Data was collected via an online self-report survey assessing thwarted belongingness, empathy, and attitudes toward culture change.

**Results:**

Whether participants experienced thwarted belongingness was not directly related to their level of support for culture change. Individuals' thwarted belongingness was indirectly and negatively associated with support for culture change, through its impact on empathy.

**Discussion:**

Taken together, the results demonstrate that cadets' experience of belongingness contributed to their level of empathy, which together predicted their support for culture change initiatives. Efforts to change the culture of the CAF may need to consider improving members' levels of belongingness and, by extension, their levels of empathy. Implications for inclusion efforts are discussed.

## 1 Introduction

The Canadian Armed Forces (CAF)'s organizational culture has been under intense scrutiny following several reports outlining the organization's lackluster responses to sexual misconduct in its ranks (e.g., Deschamps, 2015; Wells, 2021; Arbour, 2022; Coletta, 2022). A 2015 external review into sexual misconduct and harassment in the CAF found that there was a significant disconnect between the CAF ethos and values and the reality faced by its members (Deschamps, 2015). While one of the CAF's stated principal values is respecting the dignity of all people, Justice Deschamps identified an underlying sexualized and hostile culture rife with discrimination and sexual violence (e.g., Deschamps, 2015; Rosenstein et al., 2018; Richard and Molloy, 2020). In response to the external review, General Jonathan Vance, then Chief of Defence Staff (CDS), initiated and outwardly supported Operation HONOUR, with the mission to “eliminate harmful and inappropriate sexual behaviour” from the CAF (Vance, 2016; Government of Canada, 2022a). Ironically, Vance himself was later accused of having an inappropriate relationship with a subordinate (Stephenson et al., 2021a). The investigation revealed a plethora of systemic failures (e.g., Connolly, 2021; The Canadian Press, 2021); casting a harsh spotlight on the issue of sexual misconduct in the CAF and triggering a domino effect of investigations against at least 13 current and former top brass (Burke and Brewster, 2021). These events highlighted that previous attempts at culture change had been glossed over and lacked true substance (e.g., Deschamps, 2015; Coletta, 2022; Government of Canada, 2022d). The current CDS has since acknowledged the urgent need to change CAF culture, however, effective change will rely on not only systemic and legislative changes; but will also require both attitudinal and behavioral modifications by serving CAF members. To date, little is known about the factors that influence CAF members' support for culture change^1^ initiatives (for an exception see Deng et al., 2023). To contribute to the development of persuasive initiatives, this study aims to expand the nomological network surrounding attitudes toward culture change in the CAF through an investigation of how serving military members' sense of belongingness and empathy could impact their support for culture change initiatives.

Belonging has been established as a fundamental human need (Baumeister and Leary, 1995); when individuals feel that they are not included, they experience the undesirable psychological state of thwarted belongingness (Ryan and Deci, 2000). We hypothesize that the experience of thwarted belongingness could have an impact on support for culture change initiatives because, when the need to belong is unmet, it has significant repercussions for individuals' behaviors in group contexts. Specifically, thwarted belongingness inhibits group-promoting (i.e., desirable) and incites group-limiting (i.e., harmful) behaviors (Baumeister and Leary, 1995; Twenge et al., 2001; Tyler and Blader, 2003). Unmet belongingness needs could contribute to reduced empathy for others because it reduces individuals' capacity to access the emotions needed to simulate others' emotions and experiences (e.g., DeWall and Baumeister, 2006; Baumeister et al., 2007; Twenge et al., 2007). Empathy, in turn, represents an important potential mediator due to its promotion of cooperative, prosocial behavior (e.g., Eisenberg and Strayer, 1987; Batson et al., 1997). Supporting organizational culture change is prosocial (i.e., group promoting) in nature because it is designed to benefit all employees and contribute to their wellbeing and success. Thus, if individuals' belonging needs are not met, it could impact their willingness to go above and beyond for the group (e.g., Baumeister and Leary, 1995; Twenge et al., 2001). In the context of the current study, a lack of belonging could weaken social connections and psychological bonds between CAF members (e.g., Blader and Tyler, 2009) and create a circumstance whereby individuals who do not feel they belong are less concerned with the wellbeing of other CAF members, ultimately reducing their support for culture change efforts and initiatives. The proposed model aligns with several theories and empirical findings: the group engagement model suggests that when individuals' belongingness needs are unmet, their efforts for the group diminish (Tyler and Blader, 2003). In contrast, when belonging needs are satisfied, individuals will put forth greater effort for the group and have more concern for the entire group's welfare (e.g., Blader and Tyler, 2009). These ideas also align with the frustration-aggression hypothesis (e.g., Dollard et al., 1939; Berkowitz, 1989), such that feelings of exclusion and rejection promote a broad pattern of aggressive and antisocial behaviors (e.g., Twenge et al., 2001; Leary et al., 2003). The overarching aim of this research is to examine whether thwarted belongingness and empathy can collectively explain differences in attitudes toward culture change within a Canadian military sample. We begin with presenting a brief history of the CAF culture and its strides toward increasing diversity and inclusion, followed by a literature review of the variables implicated in our model, namely thwarted belongingness, empathy, and attitudes toward culture change.

### 1.1 Military culture

Investigating the CAF's current culture is a good starting point in determining the factors contributing to members' sense of belongingness, empathy, and their attitudes toward culture change. The current CAF culture is largely defined as a military culture that is sexualized (Deschamps, 2015; Eichler, 2016) and adheres to the combat, masculine-warrior paradigm (Dunivin, 1994; Greco and von Hlatky, 2020). From the CAF's early beginnings, masculinity or ‘manliness' has been linked to the identity of a warrior (e.g., Hinojosa, 2010; Lane, 2017; Pendlebury, 2020; Ferguson, 2021). The ‘warrior' ethos the CAF inculcates in its members from the beginning of their career is a prime example of the persistent reinforcement of masculine norms; the ‘warrior' is strong, relentless, brave, heroic, stoic, self-reliant, aggressive and, typically, a heterosexual male (e.g., McCristall and Baggaley, 2019; Waruszynski et al., 2019). Indeed, historically, soldiers have been encouraged to demonstrate their toughness and manliness by engaging in excessive drinking, womanizing, and brawling, all under the guise of “bonding” (Taber, 2018).

Women are at risk of experiencing negative psychological outcomes as the masculinized ‘warrior' culture inherent to the military creates a dichotomy between being a woman and being a soldier, sailor or aviator (e.g., Waruszynski et al., 2019; Pendlebury, 2020). Despite the expectation that women will suppress their femininity to assimilate, they are often mocked, belittled, and ridiculed when they adopt masculine traits (Waruszynski et al., 2019). If women do experience inclusion, they are typically perceived as “honorary men”—implying they had to be stripped of their feminine identity to be assimilated (McCristall and Baggaley, 2019).

In abandoning their femininity to fit into the military culture, women violate accepted societal gender norms. Social role theory suggests that men and women are each assigned to social roles that, in turn, dictate the differences and similarities in their behaviors (Eagly, 1987). The ideology that underscores gender differences in the military aligns with the idea that “men take life while women give it” (Duncanson, 2015, p. 232). The societal expectations of what women “should be doing”; and the demands of military service could result in women deviating from prescribed norms (e.g., Crocker et al., 1998; Brewer and Pickett, 1999; Jetten et al., 2002). This is especially problematic since the military emphasizes and idealizes agentic (e.g., strong, forceful, aggressive) norms as the standard for performance and competence (e.g., Eagly et al., 2000; Pendlebury, 2020). Indeed, even very recent systematic reviews reveal that gender stereotypes and masculine ideals persist in military forces (Reis and Menezes, 2020).

The masculine norms enforced in a male-dominated environment, such as the military, can have detrimental psychological implications for men as well (e.g., Keats, 2010; Wong et al., 2017; Milner et al., 2018). The masculinity underpinning the “ideal” CAF member is very specifically constructed and not all men can measure up to the precise performance expected by militarized masculinity (Shields et al., 2017; Taber, 2018). As such, men who do not fall within this type of masculinity also face difficulties integrating into the organization—they can be ostracized by their peers for failing to conform to expected norms and may consequently experience a thwarted sense of belongingness (e.g., Harrison and Laliberté, 2002; Taber, 2018).

Additionally, the emphasis on self-reliance in the military highlights another group of individuals who may not feel integrated in the CAF—individuals who seek mental health support in the CAF may feel they are labeled as “clients” or “patients” (Hinton et al., 2021). Since help-seeking behaviors are not aligned with the CAF's idealization of the strong, heroic, and self-reliant soldier, service members are dissuaded from seeking mental health support and, those who do seek help, may experience conflict between their military and client identities (e.g., Shields et al., 2017; Cogan et al., 2021).

Conformity to masculine norms is one of two psychological phenomena that shape how men and women experience the military organizational culture. The second is their social dominance orientation (i.e., their preference for maintaining these norms as the dominant paradigm; Deng et al., 2023). Social dominance is of relevance in military organizations as the existence of masculine norms, and a worldview that supports a social hierarchy, compounded with the lack of women in ranks, has created a gender hegemony in the CAF. Hegemonic masculinity is the maintenance of the dominant position of men in the organization and it operates on the subordination of femininity and other masculinities (e.g., Hinojosa, 2010; Duncanson, 2015). The warrior ethos cements hegemonic masculinity by privileging the military member who is male, white, able-bodied, cisgender, heterosexual, Christian, and employed in an operational role (e.g., Pendlebury, 2020; Taber, 2022). Furthermore, the hegemony marginalizes members with disabilities (e.g., Cogan et al., 2021), individuals with LGBTQIA2S+ identities, Indigenous peoples, members of visible minority groups, child rearers and bearers - or anyone who does not fit the molds prescribed by the hegemony (Taber, 2022).

A sexualized culture in the workplace is one in which sexual jokes, innuendo, teasing, and discussions are commonplace, which indicates to employees that sexualized behavior between employees is acceptable (Dekker and Barling, 1998). Importantly, sexual behavior at work is harmful to the individuals who experience it, even when those individuals report enjoying such interactions (Berdahl and Aquino, 2009). Specifically, Berdahl and Aquino (2009) discovered that despite finding sexual behavior at work fun, flattering or benign, the more sexual behavior individuals experienced, the more they withdrew from work (i.e., neglected work tasks and thought about quitting), felt less valued, experienced higher levels of depression, and used more drugs and alcohol. The existence of a sexualized culture in the CAF is evidenced through a quick review of the findings of Statistics Canada's 2018 Survey on Sexual Misconduct in the CAF. This survey revealed that 70% of Regular Force members reported they had witnessed or experienced a form of discriminatory or sexualized conduct in the 12 months preceding the survey (Statistics Canada, 2019). More specifically, 28% of women and 13% of men reported being personally targeted with sexualized or discriminatory behaviors. Overall, the prevalence of sexual assault was higher among diverse service members than those belonging to the predominant group (e.g., white, able-bodied males). Overall, women (4.3%) experienced more assaults than men (1.1%). Indigenous members (3%) reported more assaults than non-Indigenous members (1.5%), and members with disabilities (3%) disclosed more assaults than individuals without disabilities (1.5%).

### 1.2 Royal Military College culture

The Royal Military College of Canada (RMC) is a university that offers concurrent education and military training; students, who hold the rank of Naval or Officer Cadet (N/OCdt) during their studies, graduate with a university diploma and a commission allowing them to serve as officers in the CAF (Royal Military College of Canada., 2023). Canadian Military Colleges^2^ (CMC) produce an estimated 30% of CAF officers (Brulotte and Morrison, 2022) and 62% of the senior leadership (Office of the Auditor General of Canada, 2017).

Like the CAF, RMC is a male-dominated environment that has historically marginalized women and continues to endorse a masculine culture (Arbour, 2022; Deng et al., 2023). A 2019 investigation of sexual misconduct at the CMCs revealed that 68% of CMC students witnessed or experienced unwanted sexualized behavior—which closely mirrors the findings in the general CAF population reported above (Maxwell, 2020; 70%, Statistics Canada, 2019). More specifically, 52% of female students and 31% of male students reported being personally targeted with sexualized or discriminatory behaviors. Furthermore, 15% of women and 3.6% of men reported having been sexually assaulted in the 12 months preceding the survey. Scoppio et al. (2022) reported that 60% of RMC cadets indicated their colleagues made improper remarks and comments (compared with ~25% of the Regular Officer Training Plan (ROTP) cadets who were training at civilian universities). These sentiments were also echoed in Arbour's (2022) interviews with female cadets at the CMCs, who indicated the environment remained hostile and unwelcoming and that sexual misconduct and discrimination persist. Indeed, the hypermasculine and sexualized culture present in the CAF and at RMC may be hindering members' inclusion within the organization as well as their sense of belongingness.

### 1.3 Diversity, inclusion, and belongingness

In the military context, the ideas of belonging and cohesion are closely linked (Ahronson and Cameron, 2007). Cohesion is considered a broader concept that includes task cohesion (i.e., a commitment to the mission), social cohesion (i.e., social–emotional bonds among unit members), vertical cohesion (i.e., evaluations of leaders' consideration and competence) and belonging, which is a subjective evaluation of fitting in with, and belonging to the group (e.g., Dion, 2000; Hix and MacCoun, 2010). Cohesion is often cited as crucial to military functioning and the few meta-analyses and systematic reviews conducted in the military context suggest cohesion has an impact on a variety of outcomes in military populations including performance, job satisfaction, wellbeing, and retention (e.g., Oliver et al., 1999; Fors Brandebo et al., 2022).

Diversity and inclusion also have important links to belonging in the military and other contexts (e.g., Fernandez et al., 2023). Diversity refers to the presence of individuals with various demographic identities (based on factors such as language, race, gender, religion, ethnicity, sexuality, and disability; Termium, 2023), whereas inclusion refers to the respect and valuing of those differences to create a welcoming environment. Importantly, diversity efforts can be enforced through mandates and legislation. For example, allowing women entrance into all occupations in the CAF represents an effort to enhance diversity. Inclusion, however, relies on the culture and climate of the workplace and requires voluntarily actions by organizational members (Mor Barak, 2011; Hays-Thomas and Bendick, 2013; Winters, 2014). For example, treating individuals as part of the team and valuing their inputs, which cannot be mandated. Ferdman's (2017) broad definition of inclusion states, “In inclusive organizations and societies, people of all identities and many styles can be fully themselves while also contributing to the larger collective, as valued and full members” (p. 176), reflecting the idea that inclusion is focused on the fit and belonging experienced by diverse groups (Greco and von Hlatky, 2020).

In terms of diversity efforts, Canada is considered a leader in the integration of women within its armed forces (Archer, 2017) as it was among the first countries to open all occupations to women and has the highest proportions of serving women among its allies (16.3% as of April 2022, Government of Canada, 2022e). Currently, 9.6% of serving military members are visible minorities and between 2.8 and 5.5% are Indigenous (Statistics Canada, 2022a; depending on the source: Government of Canada, 2022c). Although legislation barring the entry of women, members of visible minority groups, Indigenous peoples, and members of the LGBTQIA2S+ community into the CAF have been dismantled, systemic and attitudinal barriers to full inclusion persist (e.g., Winslow and Dunn, 2002; Davis, 2022). The sheer presence of marginalized groups (i.e., the ‘add diversity and stir approach', Ely and Thomas, 2020) within the ranks is not sufficient to combat decades of deep-rooted attitudes against them (e.g., Kovitz, 2000). Thus, successful inclusion of minority groups cannot be seen in diversity statistics alone (e.g., Mor Barak et al., 2016; Davis, 2022). For example, although all occupations have been open to women for several decades, most servicewomen remain concentrated in occupations aligned with traditionally female roles including logistics (e.g., administration, supply technicians, logistics officers, and cooks) and medical (e.g., medical technicians, and nursing officers; Office of the Office of the Auditor General of Canada, 2017), highlighting that a gendered division of labor persists within the CAF (Kovitz, 2000). In addition, women and other designated group members in the CAF have struggled to advance, despite meriting promotion (Government of Canada, 2022d), mirroring civilian findings that the gender differences in performance are small but differences in rewards (such as promotion) are large, and not explained by performance differences (e.g., Joshi et al., 2015). Issues of systemic racism and white supremacy have also been identified as threats to the CAF's diversity and inclusion efforts (Government of Canada, 2022b; Pugliese, 2022a; McMaster, 2023). Together, these reports demonstrate the persistence of discrimination, hateful attitudes, and sexual misconduct within the CAF, all of which create the circumstances whereby some CAF members are likely to feel they do not belong.

### 1.4 Thwarted belongingness

From an evolutionary perspective, human beings require connections and support from others. Baumeister and Leary (1995) outline that, since the dawn of humanity, individuals have come together to reproduce, hunt, share food and resources, and protect each other. A failure to establish these connections led to death, starvation, or serious harm; as such, they suggest that creating bonds with others is an evolutionary requirement for survival leading to humans' predisposition to favor social groups and relationships. Furthermore, this innate need to belong shapes emotion and cognition and is, therefore, a powerful human motivator and fundamental need (Baumeister and Leary, 1995). Belonging requires two components to be met: a self-identity that individuals use to distinguish who they are and a group to which they can belong (Joseph et al., 2023). This human need has been widely investigated and applied in several contexts such as infant-parent relations (Bowlby, 1958, 1969), and human motivation (Maslow, 1968; Baumeister and Leary, 1995; Deci and Ryan, 2012).

The deprivation of the need to belong—thwarted belongingness—produces a psychologically painful mental state (Van Orden et al., 2012) and leads to a host of adverse physical and psychological outcomes. Beyond evident emotional distress and unhappiness, individuals deprived of social support, attachment or belongingness exhibit greater stress, maladaptive behaviors, and declining mental and physical health (Baumeister and Leary, 1995). In terms of physical outcomes, laboratory studies have linked social exclusion to physical numbness and tolerance to pain (DeWall and Baumeister, 2006; DeWall et al., 2009) and loneliness to higher urinary cortisol levels and decreased immunocompetence (Kiecolt-Glaser et al., 1984). Baumeister et al. (2002) also demonstrated that the anticipation of loneliness decreased intelligent thought. Furthermore, research indicates a significant relationship between thwarted belongingness and suicidal ideation, suicide attempts and completed suicides whereas increased connectedness to others has been linked to decreases in suicide (e.g., Joiner et al., 2006; Conner et al., 2007; Van Orden et al., 2008).

Belongingness and military culture are inextricably linked; military forces are known to value cohesion within groups to facilitate teamwork and favor operational effectiveness. Thus, military culture emphasizes the formation of strong bonds and connection between members through shared training, hardships, and experiences (e.g., Selby et al., 2010). At first glance, this suggests that military culture might promote feelings of inclusion, however, individuals that struggle to form interpersonal connections and integrate with the group may feel thwarted belongingness as they fail to connect with the highly cohesive group and experience rejection (e.g., Selby et al., 2010; Braswell and Kushner, 2012). Within the CAF's hypermasculine, sexualized culture, individuals who do not fit the warrior archetype may struggle to integrate into this cohesive group, risking being ostracized (i.e., ignored and excluded). The impact of ostracism in the military is understudied (e.g., McGraw, 2016), but civilian studies suggest ostracism may be more harmful than harassment in terms of workplace experiences (e.g., O'Reilly et al., 2015). The negative impacts of the CAF culture are not limited to individuals with marginalized identities. Indeed, the need to conform to masculine norms and the experience of sexual behavior in the workplace can have negative work-related, psychological, and wellbeing outcomes for all individuals, regardless of their group identity (e.g., Berdahl and Aquino, 2009; Keats, 2010; Shields et al., 2017; Wong et al., 2017; Milner et al., 2018).

Leary et al. (2003) found that nearly all young school shooters had felt rejected or excluded by their peers. Likewise, Twenge et al. (2001) concluded that forecasted social exclusion and rejection made participants more aggressive toward individuals who provoked them as well as neutral targets (i.e., rating them more negatively or blasting them with higher levels of aversive noise). Mirroring the notion that exclusion increases antisocial behaviors, such as aggression, social exclusion also decreased prosocial behaviors (Abdul Rashid et al., 2004). Corroborating DeWall and Baumeister's (2006) finding that exclusion leads to physical numbness, Twenge et al. (2007) demonstrated that social exclusion caused a temporary absence of emotion and a numbness to physical and emotional pain. Thwarted belongingness in the workplace has significant psychological outcomes; it undermines employees' self-concepts and impacts their self-esteem and self-efficacy (e.g., Waller, 2021). As such, when individuals feel ostracized at work, their need to belong is thwarted. This social exclusion significantly reduces individuals' empathy and trust, and can lead to counterproductive work behaviors (e.g., Twenge et al., 2007, Zhao et al., 2013; Van den Broeck et al., 2014); consequently, their performance, psychological wellbeing, and workplace contributions could be diminished.

### 1.5 Empathy

Empathy can be described as a multi-faceted, other-oriented response that encompasses both emotional and cognitive components (e.g., Davis, 2006, 2018; Vachon et al., 2014). Numerous definitions of empathy exist (see Cuff et al., 2016; Hall and Schwartz, 2019), however, we focus on two well-established aspects of empathy that are broadly recognized across various definitions: cognitive role-taking and affective reactivity (e.g., Neumann et al., 2015; Davis, 2018; Wang et al., 2020). Empathic concern is an affective phenomenon that involves either sharing the emotional state of the affected person or offering a supportive emotional response based on the situation (Davis, 2006). Perspective taking reflects the cognitive understanding of empathy and refers to accurately determining the internal emotional state of the affected person without necessarily experiencing the same emotions (Davis, 2006). Empathic concern and perspective taking (as measured by the IRI) can be combined to represent a general factor of empathy that encompasses both the affective and cognitive elements (e.g., Siu and Shek, 2005; Nicol and Rounding, 2013; Wang et al., 2020). In the present study, empathy refers to the emotional and cognitive capacity to comprehend and engage with the experiences and emotional states of others (e.g., Davis, 1983; Nicol and Rounding, 2013).

Empathy impacts attitudes and can contribute to prosocial behaviors while inhibiting antisocial behaviors (e.g., Eisenberg and Miller, 1987; Miller and Eisenberg, 1988; Batson et al., 1997, 2002). In general, prosocial behavior represents a broad set of actions defined as generally beneficial to other people by either most of society or one's social group (e.g., Penner et al., 2005). Several nuances are present in the definition above. First, prosocial behaviors are interpersonal actions involving a benefactor (the person who acts) and one or more recipients of the action. Second, what counts as prosocial within a group or society is partially determined by the circumstances and context in which the actions occur (Dovidio et al., 2017). Empathy predicts a variety of prosocial behaviors including helping others, providing resources to stigmatized groups and whistle blowing (e.g., Batson et al., 1997, 2002; Cialdini et al., 1997; Singer et al., 1998; Kamas and Preston, 2021). Considering the workplace context of the current research, empathy can also reduce sexism and sexual assaults (e.g., Berg et al., 1999; O'Donohue et al., 2003; Nicol and Rounding, 2013). For example, Rau et al. (2010) found that US Navy personnel who participated in sexual assault prevention training improved their rape knowledge, reduced their endorsement of rape myths, and increased their empathy for rape victims, which are all factors associated with the likelihood of male perpetration of sexual assault.

One major source of theoretical debate is *why* empathy predicts prosocial actions. The empathy-altruism hypothesis (e.g., Batson et al., 1991, 1997, 2015) suggests the relationship is due to altruistic (other-focused) reasons. However, a much-debated counter-argument is that empathy prompts individuals toward prosocial behavior for self-motivated reasons (e.g., Neuberg et al., 1997). Support for both altruistic and egoistic explanations exist (e.g., Cialdini et al., 1987, 1997; Batson et al., 1997), thus, for the purpose of this paper, the motivation behind empathy predicting prosocial behavior could be either self- or other-promoting. Since organizational culture change is designed to improve circumstances for all CAF members, purely altruistic motives are not required to promote positive cultural reforms. Given that empathy has been positively linked to prosocial behavior and acts as an inhibitor to antisocial behavior, a connection between empathy and supportive attitudes toward improving the CAF culture is expected.

### 1.6 Organizational culture change in the CAF

We use the following definition of culture change in the CAF: the “combined effort to realign CAF members' actions with values that reflect the Canadian society and the professional military ethos, which includes the principles of respect for the dignity of all persons, duty, loyalty, integrity, courage” (Deng et al., 2023, p. 8). The definition of culture change outlined above has several elements important to the present study. First is the alignment of values between the CAF and Canadian society. One factor influencing society's values is the changing demography of Canada. Since the 1990s, immigration has been the largest contributor to population growth in Canada. As a result, 8 million or 22% of Canadians were racialized (i.e., belonging to a visible minority group) in 2016, a proportion that is projected to grow to around 40% by 2041 (Statistics Canada, 2022b). This suggests that recruiting targets within the CAF will likely need to match the changing demographics of Canadian society more closely, because this represents the pool of potential recruits for the CAF (e.g., Mangat et al., 2020). A lack of representation by minority groups could seriously harm the CAF's operational effectiveness. Already, the CAF Regular Force is short of personnel by 8,200 (Dyson, 2023); thus, maintaining (and/or growing) the CAF will involve successful recruiting efforts targeting diverse populations (e.g., women and other minority groups). Specifically, although white men make up 39% of the Canadian labor force, they currently make up 71% of the CAF (Gallant, 2022). An overreliance on the continued recruiting of white males will not be able to support the future personnel requirements of the CAF (Pierotti, 2020). Nonetheless, effective demographic shifts within the military must involve not just greater representation of women, members of visible minority groups, and Indigenous people but also require their inclusion (e.g., Shore et al., 2011). Recruiting diverse newcomers to the CAF is not sufficient to address the changing demography in Canada (and consequently in the CAF), indeed, there is a pressing need to provide a welcoming environment for all members (Greco and von Hlatky, 2020; Pendlebury, 2020; Eyre and Matthews, 2022; Gallant, 2022).

### 1.7 Attitudes toward culture change

Culture change initiatives within the CAF are designed to advance equality for women and other minority groups and put an end to inappropriate conduct like sexual harassment (Eyre, 2021). CAF members develop attitudes toward organizational change initiatives; attitudes are evaluations of any object in terms of favorability (Eagly and Chaiken, 1993). Attitude objects can range from concrete items that exist in physical space (like people or things) to abstract concepts and social issues (Petty et al., 2003), such as culture change. Attitudes are often discussed as multicomponent constructs composed of three classes of evaluative responses: affective, cognitive, and behavioral (e.g., Rosenberg et al., 1960; Elizur and Guttman, 1976; the tripartite model, Dunham et al., 1989). For example, affective responses include being excited, satisfied, or anxious about culture change. Cognitive reactions could incorporate beliefs about the necessity or utility of proposed changes. Finally, the behavioral tendency (or instrumental reaction) refers to how individuals intend to react in response to changes in the organization (Elizur and Guttman, 1976; Dunham et al., 1989). Positive attitudes toward culture change might be more pronounced for individuals who espouse an expanded definition of the “ideal warrior” (e.g., Breede and Davis, 2020; Gregory, 2022). Corroborating this idea, Deng et al. (2023) found that female cadets supported cultural change more strongly than their male counterparts, potentially because these reforms were expected to contribute to the creation of an equal platform for women to work and perform. Aligned with this concept, the current CAF Chief Warrant Officer comes from a support (rather than an operator) occupation and their selection was based upon an enhanced process designed to ensure their values aligned with those of the CAF (Rehman, 2023). Nonetheless, negative or mixed reactions to change in organizations remain common (e.g., Piderit, 2000) for a variety of reasons such as culture change posing a psychological threat, a threat to status, and indicating the potential for disruptions to social arrangements (e.g., Dawson, 1994; Abdul Rashid et al., 2004; Erwin and Garman, 2010). To date, very few culture change initiatives in the CAF have been effectively integrated, which contributes to a sentiment that culture change is more of a buzzword than an achievable goal ( e.g., Deschamps, 2015; Duval-Lantoine and Imra-Millei, 2021; Arbour, 2022). Indeed, Arbour's (2022) external review revealed a sense of skepticism toward culture change initiatives in the CAF; members feel as though the appearance of activity is prioritized over tangible, effective efforts—in other words, culture change is seen as a form of lip service. Two divergent examples will be used to showcase the polarized reasons for which individuals could experience negative attitudes toward culture change initiatives. First, some individuals who support and long for change in the CAF but have lost confidence that this is an achievable aim. A case in point was the resignation of Lieutenant-Colonel (LCol) Eleanor Taylor. As a senior female officer in the infantry branch, and one of the CAF's most prominent women (Brewster and Everson, 2021), LCol Taylor expressed frustration toward the institution's response to its sexual misconduct crisis and its inability to hold senior officers accountable for their actions (Brewster and Everson, 2021; Stephenson et al., 2021b).

On the opposite end of the spectrum, an exemplification of negative sentiments toward culture change by individuals who do not support the need for reform in the CAF can be seen in the standing ovation given by serving military members after a speech by retired Lieutenant-General Maisonneuve which denounced both the Canadian government and military for the provision of victim apologies and the removal of historic monuments. Maisonneuve also criticized Canadian society for being entitled, and journalists for being “woke” (Pugliese, 2022b).

Both examples indicate that there is a lack of uniform support for the CAF's culture change. Deng et al. (2023) confirmed that conformity to masculine norms and social dominance orientation (i.e., the preference for maintaining masculine norms as the dominant paradigm) were two psychological phenomena that shaped attitudes toward organizational culture change at RMC. Specifically, the more that cadets conformed to masculine norms, the higher their preference for in-group dominance and out-group inequality, which together predicted less support for CAF culture change.

### 1.8 The present study

The research presented above demonstrates that the CAF's history of discriminatory practice and legislation has created a system that marginalizes groups such as women, LGBTQIA2S+ people, Indigenous people, and members of visible minority groups and consequently enables a harmful, sexualized, and hypermasculine culture. There have been many calls for the evolution of CAF culture to better reflect Canadian values; however, the actions taken by the organization to date have had limited success. For example, Madame Deschamps' (2015) third recommendation called for the establishment of an independent reporting center for sexual misconduct. Though the Sexual Misconduct Support and Resource Centre was ultimately established, it does not have control over the handling of complaints of sexual misconduct as originally suggested.

To effectively create policies and training programs aimed at achieving culture change, a greater understanding of the mechanisms surrounding members' attitudes toward culture change is required.

Past research indicates that thwarted belongingness is associated with decreased empathy and prosocial behaviors (e.g., DeWall and Baumeister, 2006; Baumeister et al., 2007; Twenge et al., 2007). This begs the exploration of how thwarted belongingness may affect members' attitudes toward culture change in the CAF. Accordingly, this study aims to expand the nomological network surrounding serving members' attitudes toward culture change. We suggest a model in which empathy acts as a mediator between individuals' feelings of thwarted belongingness and their attitudes toward culture change. Because our criterion, attitudes toward culture change, is conceptualized at the general level, we also examine our predictor (thwarted belongingness) and mediator (empathy) at the global level (e.g., Carr et al., 2003; Judge and Kammeyer-Mueller, 2012).

We posit that the experience of thwarted belongingness is important because it influences individuals' behaviors in group contexts. As thwarted belongingness impedes actions that promote the group and encourages actions that work against the group (e.g., Baumeister and Leary, 1995). Specifically, a lack of belonging could weaken social relationships and reduce psychological closeness between CAF members (e.g., Blader and Tyler, 2009) thus creating a circumstance whereby individuals whose belonging needs are thwarted are less invested in the wellbeing of other CAF members, ultimately reducing their support for culture change efforts and initiatives. Unmet belongingness needs are known to decrease empathy (e.g., DeWall and Baumeister, 2006; Twenge et al., 2007), because they reduce individuals' ability to reproduce others' emotions and experiences (e.g., DeWall and Baumeister, 2006; Baumeister et al., 2007; Twenge et al., 2007). As such, we predict the following:

*Hypothesis 1: Thwarted belongingness will be negatively correlated with empathy, such that higher levels of thwarted belonginess will be associated with lower levels of empathy*.

Empathy impacts prosocial attitudes and behaviors (e.g., Eisenberg and Miller, 1987; Miller and Eisenberg, 1988; Batson et al., 1997, 2002). Supporting culture change is considered prosocial because cultural reforms are intended to be beneficial to CAF members (e.g., Penner et al., 2005; Eyre, 2021). In addition, empathy impacts individuals' attitudes toward, and drive to act on behalf of marginalized groups (Batson et al., 2002). Thus:

*Hypothesis 2: Empathy will be positively correlated with attitudes toward culture change, such that higher levels of empathy will be associated with more support for culture change*.

We propose that the relationship between thwarted belongingness and attitudes toward culture change will be driven by its impact on empathy. Empathy represents an important potential mediator due to its promotion of cooperative, prosocial behavior (Eisenberg and Strayer, 1987; e.g., Batson et al., 1991). Thus, we also predict:

*Hypothesis 3*. *Empathy will mediate the relationship between thwarted belongingness and attitudes toward culture change, such that thwarted belongingness will be indirectly and negatively associated with support for culture change*.

## 2 Materials and methods

### 2.1 Participants

The survey invitation was sent to the Cadet Wing master email list, which includes ~1,100 Naval and Officer Cadets. One hundred eighty-one individuals consented to participate. Forty-two (23%) of those respondents did not complete most of the scale items and were removed from the data set. The final sample size of this study comprised of 139 military students. Most respondents' ages were between 18 and 21 years old (79.8% *n* = 111). There were more men (61.2%, *n* = 85) than women (36.7%, *n* = 51) and students who identified as gender non-normative (2.2%, *n* = 3). The majority (94, 67.6%) did not identify as belonging to a visible minority group. This sample is generally representative of the RMC population in terms of age, and year of study, however, the gender distribution of this sample over represents the female-to-male ratio of the ROTP population at RMC (23%; Arbour, 2022) as well as the proportion of women in the CAF (16.3%; Government of Canada, 2022e). Those belonging to visible minority groups (36.7%) also over-represents the ratio at RMC (24%; Arbour, 2022). See Table 1 for a summary of the sample's demographic characteristics.

**Table 1:** Sociodemographic characteristics of participants.

	**Sample population (*****n*** = **139)**	
**Characteristics**	***n***	***%***
**Gender**		
Female	51	36.7
Male	85	61.2
Non-conforming	3	2.1
**Age**		
17	3	2.2
18–21	111	79.8
22+	25	18.0
**Member of visible minority group**		
Yes	45	32.4
No	94	67.6
**Year of study**		
1	29	20.9
2	48	34.5
3	25	18.0
4	37	26.6

### 2.2 Procedure

This study received approval from the Research Ethics Board at RMC. All the participants of this study were recruited by a bilingual (i.e., French and English) email directly distributed to each military student via the Cadet Wing master email list on Webmail—the primary method of professional communication at RMC. This email was composed of an overview of the research and the link to the questionnaire mounted on SurveyMonkey. Participants who gave their consent completed the measures, followed by a demographics section. Items for all measures, except the demographic questionnaire, were presented in random order. The measures themselves were also presented in random order. Finally, a debriefing page explained the full nature of the study.

### 2.3 Measures

#### 2.3.1 Thwarted belongingness

The 9-item Thwarted Belongingness subscale of the Interpersonal Needs Questionnaire (Van Orden et al., 2012) was administered to participants. This measure was scored using a 5-point Likert scale on which participants rated each item on a scale of (*1* = *not at all true for me*) to (*5* = *very true for me*). Higher scores indicated greater levels of thwarted belongingness and the scale showed an internal consistency of α = 0.87.

#### 2.3.2 Empathy

To assess empathy at an individual level, two subscales of Davis' (1980) Interpersonal Reactivity Index (seven items reflecting Empathic Concern and seven items representing Perspective Taking) were administered.^3^ Higher scores reflect more of the construct. The two scales summed together represent an index of general empathy (see also Nicol and Rounding, 2013; Wang et al., 2020). This measure was rated on a 5-point Likert scale where participants rated each item from (*1* = *strongly disagree*) to (*5* = *strongly agree*). Higher scores indicated greater levels of empathy and the index showed an internal consistency of α = 0.78.

#### 2.3.3 Attitude toward CAF culture change

An 11-item measure was used to examine support for cultural change in the CAF. We used Deng et al.'s (2023) 10-item scale (adapted from Dunham et al., 1989). To make the scale more gender inclusive, we added the item “all CAF members will benefit from culture change”. In total, five items evaluated personal liking and commitment to culture change and six items tapped into one's general view of the institutional consequences of culture change (Dunham et al., 1989; Deng et al., 2023). Participants were asked to read a brief description defining culture change in the CAF prior to filling out the scale. Participants reported on a 7-point Likert scale from 1 (*strongly disagree*) to 7 (*strongly agree*). This scale had a high internal consistency of α = 0.89. Higher aggregated scores indicated greater acceptance of culture change in the CAF.

#### 2.3.4 Demographic information

All participants answered demographic questions pertaining to their age, gender, year of study and membership in a minority group. This data was used to help define the sample, determine if it was representative of the RMC student body, and identify the possibility of group differences.

## 3 Results

### 3.1 Preliminary analysis

All analyses were computed using SPSS 28. Attributes such as gender, and membership in a minority group may contribute to levels of belonging, empathy, and support for culture change (e.g., Walton and Cohen, 2011; Kamas and Preston, 2021; Deng et al., 2023). Therefore, independent *t*-tests were conducted to determine if significant differences based on gender or ethnicity were present in the independent variable, mediator, and dependent variable. Individuals who reported belonging to a visible minority group did not differ than those who did not in terms of belonging *t*_(138)_ = 1.365, *p* = 0.175, empathy *t*_(137)_ = 0.496, *p* = 0.621 or attitudes toward culture change *t*_(137)_ = 0.980, *p* = 0.329 There were also no differences between male and female participants in terms of their level of belongingness *t*_(135)_ = −0.215, *p* = 0.415. Female students scored significantly higher than male students on empathy, *t*_(134)_ = −2.529, *p* = 0.006, and this difference corresponded to a moderate effect size, Cohen's *d* = −0.448 (using the cut-offs provided by Lovakov and Agadullina, 2021). Women were also more supportive of organizational culture change than men *t*_(134)_ = −3.669, *p* ≤ 0.001, and this difference corresponded to a large effect size, Cohen's *d* = −0.653. See Table 2 for all correlations. As a result of our independent samples *t*-tests, only gender was entered as a covariate in our indirect effects analysis (see Table 3). Two-tailed Pearson correlations with bootstrapping were calculated to test Hypotheses 1 and 2 which explored the relationships between thwarted belongingness, empathy, and attitudes toward CAF culture change.

**Table 2:** Descriptive statistics and correlations for all study variables.

**Variable**	***M***	***SD***	**1**	**2**	**3**	**4**
1. Gender	1.38	0.52	—			
2. Thwarted belongingness	2.32	0.87	0.062	(0.87)		
3. Empathy	3.54	0.50	0.196^*^	−0.198^*^	(0.78)	
4. Attitudes toward culture change	4.40	0.82	0.301^*^	−0.102	0.433^*^	(0.89)

Reliabilities are on the diagonal in parentheses.

^*^p < 0.05 (two-tailed).

**Table 3:** Effects of thwarted belongingness on attitudes toward culture change through empathy *N* = 139.

**Predictors**	**Mediator** = **empathy**				**DV** = **attitudes toward culture change**			
	***b***	***SE***	**95% CI**		***b***	***SE***	**95% CI**	
			**LLCI**	**ULCI**			**LLCI**	**ULCI**
Thwarted belongingness	−0.1196	0.0479	−0.2142	−0.0249	−0.0302	0.0732	−0.1749	0.1146
Empathy					0.6418	0.1305	0.3836	0.9001
**Indirect effect**			
Thwarted belongingness through empathy					−0.0767	0.0344	−0.1515	−0.0179

Hypothesis 3 constituted a test of mediation and was tested using procedures implemented in PROCESS v.4 using Model 4 (http://www.afhayes.com; Hayes, 2022, 2023). A linear regression equation was estimated in which the dependent variable (attitudes toward culture change) was regressed on empathy as the mediator, and thwarted belongingness as the independent variable, gender was entered as a covariate. Bias corrected 95% confidence intervals (CI) based on 5,000 bootstrapped resamples were used to determine the statistical significance of the effects (MacKinnon et al., 2004). The indirect effect was deemed significant when the bias-corrected CI for an indirect effect did not include zero. Unstandardized OLS regression coefficients are reported.

### 3.2 Hypothesis testing

Supporting our first hypothesis, there was a significant negative correlation between thwarted belongingness and empathy, *r*_(136)_ = −0.198, *p* = 0.02. Empathy was positively related to higher acceptance for culture change, as predicted by our second hypothesis, *r*_(136)_ =0.433, *p* < 0.001.

**Figure 1:**
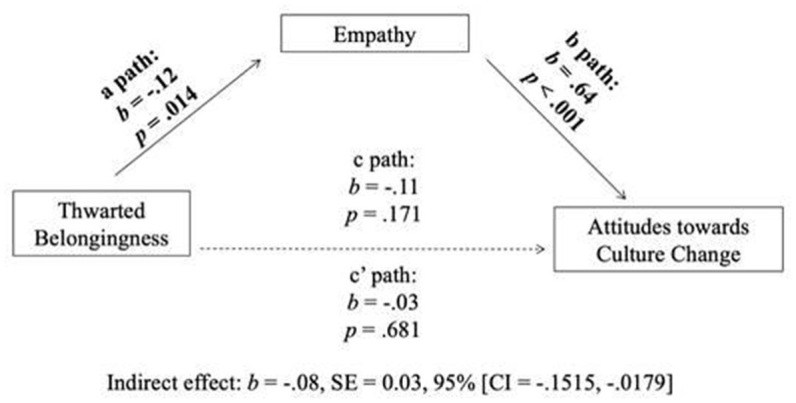
Effects of thwarted belongingness on attitudes toward culture change through empathy.

We posited that thwarted belongingness would affect attitudes toward culture change through empathy. Confirming hypothesis 3, there was an indirect, negative, relationship between thwarted belongingness and attitudes toward culture change through empathy. Specifically, the total effects showed that individuals' levels of thwarted belongingness did not significantly predict attitudes toward culture change (*b* = −0.107, *p* =0.18) CI [−0.26, 0.05]. The overall indirect effect through empathy was significant, (*b* = −0.077), 95% CI [−0.15, −0.02]. Finally, the direct effect between thwarted belongingness and attitudes toward culture change was not significant, *b* = −0.03, *p* = 0.68, CI [-0.18, 0.11]. See Figure 1, Table 3 for the results of the indirect effects model.

Gender was a significant predictor of both empathy and attitudes toward culture change, however, the hypothesized model remained significant whether or not gender was included as a covariate in the analyses.

## 4 Discussion

This study was based on the assumption that individuals' thwarted belongingness and empathy would be important predictors of their attitudes toward culture change. As hypothesized, empathy had an indirect effect on the association between thwarted belongingness and attitudes toward culture change.

Importantly, in this study, whether participants experienced thwarted belongingness was not directly related to their level of support for culture change. Individuals' thwarted belongingness was related to attitudes toward culture change, through its impact on empathy. Taken together, the results demonstrate that cadets' experience of belongingness contributed to their level of empathy, which together predicted their support of culture change initiatives.

In our sample, male military students scored significantly lower than female cadets did on both empathy and support for culture change. These results support previous literature indicating that women tend to display more empathy than men (e.g., Cuddy et al., 2011; Willer et al., 2015; Kamas and Preston, 2021), and that male cadets displayed lower levels of support for culture change than their female counterparts (Deng et al., 2023). These results may further underscore Deng et al.'s (2023) finding that masculine norm conformity has an important impact on how male and female students experience the organizational hierarchy at RMC. For example, female cadets may support culture change more strongly because changes could mean they are able to perform for women to work and perform without having to measure up to the masculine standards enmeshed in the warrior identity. Conversely, male cadets, particularly those whose identities align with the warrior ideal, may be reluctant to support changes to the organizational hierarchy that could threaten their privileged standing (Dawson, 1994; Deng et al., 2023).

We expected that individuals from marginalized groups (e.g., women and members of visible minority groups; Walton and Cohen, 2007) might experience less belonging within the CAF and RMC. However, in our sample, levels of thwarted belongingness did not differ between men and women, or between individuals who identified as belonging to a visible minority group (vs. those who did not) as measured by the Interpersonal Needs Questionnaire (Van Orden et al., 2012). There are several reasons why this may have occurred, first, the demographic composition of our study sample over-represented individuals who identified as women and members of visible minority groups, which could have impacted our findings. Importantly, the fact that women and members of visible minority groups are slightly over-represented at RMC (compared to the CAF) may offer them a greater sense of belonging due to their representation rates approaching those considered a “critical mass” (Kanter, 1977). Arguably, (had our sample been more closely aligned with the demographics of the CAF, we may have been able to detect differences between groups. Second, because the Interpersonal Needs Questionnaire asks about general belonging, we may not have directly assessed a sense of belonging to the CAF or within RMC. Our logic was that marginalized groups might experience less belonging within the military organization, but not necessarily with their friends and family. Finally, other research suggests that younger men experience more loneliness than women (e.g., Koenig et al., 1994; Barreto et al., 2021). Therefore, finding no difference in this sample may support the notion that the masculine norms embedded within RMC and the CAF led to underreporting of thwarted belongingness for the men in our sample (Cramer and Neyedley, 1998; e.g., Chu et al., 2017).

### 4.1 Conceptual and practical implications

These findings have conceptual implications and help to further our understanding of attitudes toward culture change. There was an indirect impact of thwarted belongingness through empathy on attitudes toward culture change. From a practical standpoint, the CAF may wish to directly increase empathy levels in their members as a method to increase overall investment in and support of culture change initiatives. Empathy levels can be influenced, and thus, improved—training can lead to a decline in aggressive behaviors and an increase in prosocial behaviors (Konrath et al., 2010). Empathy increasing training programs may help the CAF attain its culture change aims, as individuals who demonstrate empathic concern and perspective taking are more capable of understanding experiences and offering assistance (e.g., Batson et al., 2002; Lee et al., 2019). Encouraging empathy for victims is also an important part of training programs targeted to reduce sexual assault (e.g., Schewe, 2002). The CMCs may also represent a fertile ground for belonging interventions (e.g., Walton and Cohen, 2011; Murphy M. C. et al., 2020; Walton and Brady, 2020; Walton et al., 2023). Walton and Cohen (2011), Walton and Brady (2020), Walton et al. (2023) have found success in interventions designed to enhance belonging in racially marginalized college students, leading to higher GPAs and self-reported health, wellbeing, and life satisfaction. Gilken and Johnson (2019) found a belonging intervention was effective for community college students, increasing social and academic relatedness. Interventions aimed specifically at mitigating thwarted belongingness tend to focus on reducing the risk for suicide. Short et al. (2019) demonstrated that brief, computerized belongingness interventions among veterans can effectively decrease feelings of thwarted belongingness. As such, implementing programs or training aimed at increasing the sense of belongingness in the organization could help mitigate feelings of thwarted belongingness in the military, and contribute to reduced risk for suicide. Based on the conducted study, this decrease in thwarted belongingness could consequently lead to an increased propensity to display empathy, which is an important contributor to CAF members' capacity to support initiatives related to culture change.

Military leaders may have a particularly important role to play in shifting organizational norms to create a climate characterized by respect and professionalism (e.g., Sadler et al., 2017). Specifically, military leaders (who are predominantly male) can outwardly support the advancement of women and other marginalized groups, which in turn can influence the attitudes and behaviors of other men (e.g., Drury and Kaiser, 2014; Cheng et al., 2019). Leaders who support military culture change efforts must learn to communicate the importance and value of every military member regardless of their gender, race, sexual orientation, occupation, and rank (Thomas and Ely, 1996; Madsen et al., 2020). This may require a reconceptualization of what constitutes the ideal “warrior” in modern times (e.g., Waruszynski et al., 2019; Breede and Davis, 2020; Gregory, 2022), and an understanding that it is not by embracing all that is masculine that individuals advance to the highest levels of the military (Hinojosa, 2010). Instead, service members who best reflect the CAF's values of loyalty, integrity, and courage, excellence, inclusion, and accountability, as stated in the updated CAF ethos, *Trusted to Serve*, (Government of Canada, 2022b) should be selected as the future leaders of the CAF.

In a similar vein, dismantling and reconceptualizing the current understanding of what it means to be a warrior may be pertinent to the CAF's current inclusion efforts. The findings of this study demonstrate that the women and visible minority participants surveyed did not experience a lesser sense of general belonging. However, these individuals may still face difficulties in assimilating the organizational norms, and warrior culture in the CAF (e.g., Pendlebury, 2020; Taber, 2022); a conclusion we cannot make based on our data. Nonetheless, redefining the CAF's culture and identity would allow members of all groups to feel welcome, valued, respected and, therefore, contribute to a sense of belonging. The relationships established between belonging, empathy, and attitudes toward culture change may be leveraged to create a more inclusive environment.

### 4.2 Limitations and future research

This study has limitations that must be considered. Firstly, all data was collected using a cross-sectional design, therefore, the relationships between thwarted belongingness, empathy, and attitudes toward culture change should not be interpreted causally. This may limit the interpretability of this study as mediation analyses rely on a causal sequence of events (e.g., MacKinnon and Pirlott, 2015). Indeed, it is possible that this study could reflect elements of reverse causality. Mono-method bias is also present due to the utilization of only self-report data. Conway and Lance (2010) provided several suggestions for how authors could address common method concerns in their research. First, when considering the factors analyzed in this study, self-report measures are most appropriate. Specifically, experiences of thwarted belongingness, empathy, and attitudes toward culture change are all personal judgments based on individuals' perceptions, that another source may have difficulty evaluating objectively. There is also a lack of conceptual overlap in the items for thwarted belongingness, empathy, and attitudes toward culture change. Therefore, it is unlikely that common method bias explains our findings. Nonetheless, future research using multiple methods would provide further insights. Lastly, the response rate is a limitation in this study. The study was made available to the entire cadet population at RMC (~1,100 students), however only 139 cadets opted to respond to most of the survey items. This means that the study may not have accurately represented the overall sentiment at the college.

Moreover, as identified above, RMC is a unique military unit; and the military members in the sample were students mostly between the ages of 18 to 21, which is representative of the population at RMC. However, this sample does not accurately characterize the CAF's working population in terms of age and gender; specifically, this study over-represents women at RMC (23%) by 14% percent and women in the CAF (16.3%) by 21% (Arbour, 2022; Government of Canada, 2022e). Our sample also over-represents individuals who identify as being part of a visible minority group (36.7% vs. 24% in the RMC population, Arbour, 2022). As such, these findings may lack generalizability to the CAF, other militaries, or in other male-dominated industries, where harassment and discrimination are also highly present (e.g., STEM, Moser and Branscombe, 2021). The associations between thwarted belongingness, empathy, and attitudes toward culture change should be investigated with a larger population of serving members to gain a more comprehensive understanding of attitudes toward culture change in the CAF more broadly.

There are several avenues for future research. For example, examining a different predictor variable with a high degree of alignment with our proposed model, such as the Warrior Identity Scale (WIS, Lancaster et al., 2018) or the Military Professional Identity Scale (NPIS; Johansen et al., 2013) might lead to more clarity on the impact of not belonging within the military. The subscales of the WIS include constructs such as military connection, identity commitment, military as a family, and military centrality which are all related to members' sense of belongingness to the organization (Lancaster et al., 2018). Using such scales may also provide insights on the impacts of the military's socialization and indoctrination processes on individuals' sense of belonging.

Further research is needed to fully understand the role of masculine norm conformity on men's capacity for empathy (e.g., Gabbiadini et al., 2016; Chu and Gilligan, 2019). It is also possible that military service impacts individuals' ability to show empathy, though very little empirical data to support this notion exists. Brænder and Andersen (2013) found that soldiers reported less compassion for others after serving in Afghanistan. Furthermore, should empathy training programs be considered, it will be important to monitor their impact. Specifically, we may consider how much empathy is appropriate for a CAF member to display and whether too much empathy runs the risk of venturing into emotional contagion. Based on the highly masculine environment present in the CAF, there is little reason to expect empathy training programs are likely to result in harmful experiences such as compassion fatigue (e.g., Doherty et al., 1995). In fact, recent research suggests that emotional contagion from leaders (such as empathy, understanding, and support) can strengthen the bonds between military unit members (Abdurachman, 2022). Nonetheless, such training programs should maintain the self-other distinction (Coll et al., 2017); empathy focuses on recognizing another's distress rather than adopting that distress, which could be the case if one experiences emotional contagion. While emotional contagion could result in personal distress (Coll et al., 2017), empathy is expected to bring about prosocial behavior (Eisenberg and Miller, 1987; Batson et al., 1997; De Waal, 2008).

Additionally, we did not examine the potential impact of the individual facets of empathy and instead focused on a global representation of empathy. Because our criterion, attitudes toward culture change, was conceptualized at the general level, it would not have been appropriate to examine the facet level of empathy in this study (e.g., Carr et al., 2003; Judge and Kammeyer-Mueller, 2012). Nonetheless, future research could consider more granular operationalizations of thwarted belongingness, empathy, and attitudes toward culture change, which could help determine which aspects of empathy and belongingness would be most relevant for future organizational interventions.

Finally, a future study using a longitudinal design could be particularly relevant in determining the role RMC plays in the development of attitudes toward culture change as well as the impact the institution may have on thwarted belongingness. Since RMC produces a high proportion of CAF officers and senior CAF leaders, it is imperative that a comprehensive understanding of its impact on the attitudinal and behavioral development of future CAF leaders. A longitudinal study conducted by Nicol et al. (2007) at RMC indicated an increase in N/OCdt' social dominance orientation—the desire to establish and maintain a hierarchical social structure—between their first year and fourth year. Furthermore, this finding was attributed to the socialization processes within RMC and the CAF, thus legitimizing the notion that the current climate within the armed forces and the military colleges can work against culture change efforts.

First, going beyond empathy, research should identify other paths through which thwarted belongingness affect attitudes toward culture change. In line with the group value model and sociometer theory, thwarted belongingness could promote other group-limiting reactions beyond empathy (Leary et al., 1995; Blader and Tyler, 2009). For example, if you do not feel you belong, you may derogate individuals in the group (i.e., the military organization) to protect yourself from internalizing the negative impact of not being accepted (Leary et al., 1995; Tyler and Blader, 2003). Thus, future research should examine other factors related to thwarted belongingness that may also hinder support for culture change.

To conclude, the aim of this study was to increase the current understanding of factors that can promote or hinder the CAF's culture change efforts to contribute to the development of effective initiatives and strategies. Importantly, this study enhanced our understanding of the nomological network surrounding military culture change. Military organizations interested in promoting culture change, should consider the impact of two variables that can be influenced: empathy and belongingness.

## Data availability statement

The raw data supporting the conclusions of this article will be made available by the authors, without undue reservation.

## Ethics statement

This study involving humans was approved by the RMC Research Ethics Board. The study was conducted in accordance with local legislation and institutional requirements. The participants provided their informed consent to participate in this study.

## Author contributions

ÉG: Writing – review & editing, Writing – original draft. CS: Writing – review & editing, Writing – original draft, Supervision, Methodology, Funding acquisition, Formal analysis, Conceptualization. EH: Writing – original draft.

## Footnotes

^1^Within the CAF, terms such as cultural reform and cultural evolution are also employed. We have selected the term culture change as it is the only one of these terms currently recognized in Termium Plus (Termium, 2023), the Government of Canada's terminology and linguistic data bank.

^2^The Canadian Military Colleges include the Royal Military College of Canada in Kingston, Ontario and the Collège royal miltiare de Saint-Jean in Saint-Jean-sur-Richelieu, Quebec.

^3^We chose to use only two of the 4 IRI facets because use of all four facets does not appear to be statistically appropriate based on the measurement models reported in numerous studies (e.g., Siu and Shek, 2005; Garcia-Barrera et al., 2017; Lucas-Molina et al., 2017; Murphy B. A. et al., 2020).

## References

[B1] Abdul Rashid Z. A. Sambasivan M. Rahman A. A. (2004). The influence of organizational culture on attitudes toward organizational change. Leadersh. Organ. Dev. J. 25, 161–179. 10.1108/01437730410521831

[B2] Abdurachman D. (2022). Leaders' emotional contagion: unveiling the catalyst for soldiers' morale, adaptability, and unit cohesion in military settings. Int. J. eBus. eGovernment Stud. 14, 429–451. 10.34109/ijebeg.202214121

[B3] Ahronson A. Cameron J. E. (2007). The nature and consequences of group cohesion in a military sample. Milit. Psychol. 19, 9–25. 10.1080/08995600701323277

[B4] Arbour L. (2022). Report of the Independent External Comprehensive Review of the Department of National Defence and the Canadian Armed Forces. Available online at: https://www.canada.ca/en/department-national-defence/corporate/reports-publications/report-of-the-independent-external-comprehensive-review.html (accessed August 31, 2023).

[B5] Archer E. M. (2017). Women, Warfare and Representation: American Servicewomen in the Twentieth Century. London: Bloomsbury Academic.

[B6] Barreto M. Victor C. Hammond C. Eccles A. Richins M. T. Qualter P. (2021). Loneliness around the world: Age, gender, and cultural differences in loneliness. Pers. Individ. Dif. 169, 110066. 10.1016/j.paid.2020.110066 33536694 PMC7768187

[B7] Batson C. D. Batson J. G. Slingsby J. K. Harrell K. L. Peekna H. M. Todd R. M. (1991). Empathic joy and the empathy-altruism hypothesis. J. Pers. Soc. Psychol. 61, 413–426. 10.1037/0022-3514.61.3.413 1941512

[B8] Batson C. D. Chang J. Orr R. Rowland J. (2002). Empathy, attitudes, and action: can feeling for a member of a stigmatized group motivate one to help the group? Pers. Soc. Psychol. Bull. 28, 1656–1666. 10.1177/014616702237647

[B9] Batson C. D. Lishner D. A. Stocks E. L. (2015). “The empathy—Altruism hypothesis,” in The Oxford Handbook of Prosocial Behavior, eds. D. A. Schroeder, and W. G. Graziano (Oxford: Oxford University Press), 259–281.

[B10] Batson C. D. Polycarpou M. P. Harmon-Jones E. Imhoff H. J. Mitchener E. C. Bednar L. L.. (1997). Empathy and attitudes: can feeling for a member of a stigmatized group improve feelings toward the group?. J. Pers. Soc. Psychol. 72, 105–118. 10.1037/0022-3514.72.1.105 9008376

[B11] Baumeister R. F. Brewer L. E. Tice D. M. Twenge J. M. (2007). Thwarting the need to belong: understanding the interpersonal and inner effects of social exclusion. Soc. Personal. Psychol. Comp. 1, 506–520. 10.1111/j.1751-9004.2007.00020.x

[B12] Baumeister R. F. Leary M. R. (1995). The need to belong: desire for interpersonal attachments as a fundamental human motivation. Psychol. Bull. 117, 497–529. 10.1037/0033-2909.117.3.497 7777651

[B13] Baumeister R. F. Twenge J. M. Nuss C. K. (2002). Effects of social exclusion on cognitive processes: anticipated aloneness reduces intelligent thought. J. Personal. Social Psychol. 83, 817–827. 10.1037/0022-3514.83.4.817 12374437

[B14] Berdahl J. L. Aquino K. (2009). Sexual behavior at work: Fun or folly? J. Appl. Psychol. 94, 34–47. 10.1037/a0012981 19186894

[B15] Berg D. R. Lonsway K. A. Fitzgerald L. F. (1999). Rape prevention education for men: The effectiveness of empathy-induction techniques. J. Coll. Stud. Dev. 40, 219–234.

[B16] Berkowitz L. (1989). Frustration-aggression hypothesis: examination and reformulation. Psychol. Bull. 106, 59–73. 10.1037/0033-2909.106.1.59 2667009

[B17] Blader S. L. Tyler T. R. (2009). Testing and extending the group engagement model: linkages between social identity, procedural justice, economic outcomes, and extrarole behavior. J. Appl. Psychol. 94, 445–464. 10.1037/a0013935 19271800

[B18] Bowlby J. (1958). The nature of the child's tie to his mother. In. J. Psychoanalys. 39, 350–373. 13610508

[B19] Bowlby J. (1969). “Attachment and loss: Vol.1,” in Attachment (New York: Basic Books).

[B20] Brænder M. Andersen L. B. (2013). Does deployment to war affect public service motivation? A panel study of soldiers before and after their service in Afghanistan. Public Adm. Rev. 73, 466–477. 10.1111/puar.12046

[B21] Braswell H. Kushner H. I. (2012). Suicide, social integration, and masculinity in the U.S. military. Soc. Sci. Med. 74, 530–536. 10.1016/j.socscimed.2010.07.031 21036443

[B22] Breede H. C. Davis K. D. (2020). “Do you even pro, bro? Persistent testing of warrior identity and the failure of cohesion,” in Why We Fight: New Approaches to the Human Dimensions of Warfare, eds. R. C. Engen, H. C. Breede, and, A. English (Montreal QC: McGill-Queen's University Press), 119.

[B23] Brewer M. B. Pickett C. L. (1999). “Distinctiveness motives as a source of the social self,” in The Psychology of the Social Self, in eds. T. R. Tyler, R. M. Kramer and O. P. John (Mahwah: Lawrence Erlbaum Associates Publishers), 71–87.

[B24] Brewster M. Everson K. (2021). “Senior female officer quits Canadian Forces, says she's ‘sickened' by reports of sexual misconduct,” in CBC News. Available online at: https://www.cbc.ca/news/politics/anada-taylor-canadian-forces-sexual-misconduct-1.5952618 (accessed August 31, 2023).

[B25] Brulotte B. Morrison T. (2022). “In defence of Canada's military colleges,” in The Hill Times. Available online at: https://www.hilltimes.com/story/2022/06/15/in-defence-of-canadas-military-colleges/270795/ (accessed August 31, 2023).

[B26] Burke A. Brewster M. (2021). “A military in crisis: Here are the senior leaders embroiled in sexual misconduct cases,” in CBC News. Available online at: https://www.cbc.ca/news/politics/sexual-misconduct-military-senior-leaders-dnd-caf-1.6218683 (accessed August 31, 2023).

[B27] Carr J. Z. Schmidt A. M. Ford J. K. DeShon R. P. (2003). Climate perceptions matter: a meta-analytic path analysis relating molar climate, cognitive and affective states, and individual level work outcomes. J. Appl. Psychol. 88, 605. 10.1037/0021-9010.88.4.605 12940402

[B28] Cheng S. K. Ng L. C. Traylor A. M. King E. B. (2019). Helping or hurting?: Understanding women's perceptions of male allies. Personnel Assessm. Deci. 5, 44–54. 10.25035/pad.2019.02.006

[B29] Chu C. Buchman-Schmitt J. M. Stanley I. H. Hom M. A. Tucker R. P. Hagan C. R.. (2017). The interpersonal theory of suicide: a systematic review and meta-analysis of a decade of cross-national research. Psychol. Bull. 143, 13131345. 10.1037/bul000123 29072480 PMC5730496

[B30] Chu J. Y. Gilligan C. (2019). Boys' development in a new era of APA guidelines. Men Masc. 22, 909–913. 10.1177/1097184X19874871

[B31] Cialdini R. B. Brown S. L. Lewis B. P. Luce C. Neuberg S. L. (1997). Reinterpreting the empathy–altruism relationship: when one into one equals oneness. J. Pers. Soc. Psychol. 73, 481–494. 9294898

[B32] Cialdini R. B. Schaller M. Houlihan D. Arps K. Fultz J. Beaman A. L. (1987). Empathy-based helping: Is it selflessly or selfishly motivated? J. Pers. Soc. Psychol. 52, 749–758. 3572736 10.1037//0022-3514.52.4.749

[B33] Cogan A. M. Haines C. E. Devore M. D. (2021). Intersections of US military culture, hegemonic masculinity, and health care among injured male service members. Men Masc. 24, 468–482. 10.1177/1097184X19872793

[B34] Coletta A. (2022). “Sexual misconduct report finds Canadian military culture ‘deficient',” in The Washington Post. Available online at: https://www.washingtonpost.com/world/2022/05/30/anada-armed-forces-sexual-misconduct-report/ (accessed August 31, 2023).

[B35] Coll M. P. Viding E. Rütgen M. Silani G. Lamm C. Catmur C.. (2017). Are we really measuring empathy? Proposal for a new measurement framework. Neurosci. Biobehav. Rev. 83, 132–139. 10.1016/j.neubiorev.2017.10.009 29032087

[B36] Conner K. Britton P. Sworts L. Joiner T. (2007). Suicide attempts among individuals with opiate dependence: the critical role of felt belonging. Addict. Behav. 32, 1395–1404. 10.1016/j.addbeh.2006.09.012 17097813

[B37] Connolly A. (2021). “TIMELINE: The Canadian Forces sexual misconduct crisis,” in Global News. Available online at: https://globalnews.ca/news/7883717/anadan-forces-sexual-misconduct-timeline/ (accessed August 31, 2023).

[B38] Conway J. M. Lance C. E. (2010). What reviewers should expect from authors regarding common method bias in organizational research. J. Bus. Psychol. 25, 325–334. 10.1007/s10869-010-9181-6

[B39] Cramer K. M. Neyedley K. A. (1998). Sex differences in loneliness: the role of masculinity and femininity. Sex Roles 38, 645–653.

[B40] Crocker J. Major B. Steele C. (1998). “Social stigma,” in The Handbook of Social Psychology, eds. D. T. Gilbert, S. T. Fiske, & G. Lindzey (New York: McGraw-Hill), 504–553.

[B41] Cuddy A. J. Glick P. Beninger A. (2011). The dynamics of warmth and competence judgments, and their outcomes in organizations. Res. Organizat. Behav. 31, 73–98. 10.1016/j.riob.2011.10.004

[B42] Cuff B. M. Brown S. J. Taylor L. Howat D. J. (2016). Empathy: a review of the concept. Emot. Rev. 8, 144–153. 10.1177/1754073914558466

[B43] Davis K. D. (2022). Socio-cultural dynamics in gender and military contexts: Seeking and understanding change. J. Military, Veteran and Family Health. 8, 66–74. 10.3138/jmvfh-2021-0088

[B44] Davis M. H. (1980). A multidimensional approach to individual differences in empathy. JSAS Catalog Sel. Docum. Psychol. 10, 85.

[B45] Davis M. H. (1983). The effects of dispositional empathy on emotional reactions and helping: a multidimensional approach. J. Pers. 51, 167–184. 10.1111/j.1467-6494.1983.tb00860.x

[B46] Davis M. H. (2006). “Empathy,” in Handbook of the Sociology of Emotions. Handbooks of Sociology and Social Research, eds. J. E. Stets, and J. H. Turner, J.H (Boston, MA: Springer).

[B47] Davis M. H. (2018). Empathy: A Social Psychological Approach. London: Routledge.

[B48] Dawson P. (1994). Organizational Change: A Process Approach. London: Paul Chapman

[B49] De Waal F. B. (2008). Putting the altruism back into altruism: the evolution of empathy. Annu. Rev. Psychol. 59, 279–300. 10.1146/annurev.psych.59.103006.093625 17550343

[B50] Deci E. L. Ryan R. M. (2012). “Self-determination theory,” in Handbook of Theories of Social Psychology, eds. P. A. M. Van Lange, A. W. Kruglanski, and E. T. Higgins (Thousand Oaks: Sage Publications Ltd), 416–436.

[B51] Dekker I. Barling J. (1998). Personal and organizational predictors of workplace sexual harassment of women by men. J. Occup. Health Psychol. 3, 7–18. 9552268 10.1037/1076-8998.3.1.7

[B52] Deng M. E. Nicol A. A. Suurd Ralph C. (2023). Masculine conformity and social dominance's relation with organizational culture change. Armed Forces Soc. 10.1177/0095327X231178522

[B53] Deschamps M. (2015). External Review into Sexual Misconduct and Sexual Harassment in the Canadian Armed Forces. Available online at: https://www.canada.ca/en/department-national-defence/corporate/reports-publications/sexual-misbehaviour/external-review-2015.html (accessed August 31, 2023).

[B54] DeWall C. N. Baumeister R. E. Masicampo E. J. (2009). “Rejection: Resolving the paradox of emotional numbness after exclusion,” in Handbook of Hurt Feelings in Close Relationships (Cambridge, UK: Cambridge University Press), 123–141. 10.1017/CBO9780511770548.008

[B55] DeWall C. N. Baumeister R. F. (2006). Alone but feeling no pain: effects of social exclusion on physical pain tolerance and pain threshold, affective forecasting, and interpersonal empathy. J. Personal. Soc. Psychol. 91, 1–15. 10.1037/0022-3514.91.1.1 16834476

[B56] Dion K. L. (2000). Group cohesion: From” field of forces” to multidimensional construct. Group Dynam. 4, 7.

[B57] Doherty R. W. Orimoto L. Singelis T. M. Hatfield E. Hebb J. (1995). Emotional contagion: gender and occupational differences. Psychol. Women Q. 19, 355–371.

[B58] Dollard J. Doob L. Miller N. Mowrer O. Sears R. (1939). Frustration and Aggression. New Haven: Yale University Press.

[B59] Dovidio J. F. Piliavin J. A. Schroeder D. A. Penner L. A. (2017). The Social Psychology of Prosocial Behavior. London: Psychology Press. 10.1146/annurev.psych.56.091103.070141 15709940

[B60] Drury B. J. Kaiser C. R. (2014). Allies against sexism: the role of men in confronting sexism. J. Social Issues. 70, 637–652. 10.1111/josi.12083

[B61] Duncanson C. (2015). Hegemonic masculinity and the possibility of change in gender relations. Men Masc. 18, 231–248. 10.1177/1097184X15584912

[B62] Dunham R. B. Grube J. A. Gardner D. G. Cummings L. L. Pierce J. L. (1989). “The development of an attitude toward change instrument”, in *Paper Presented at the Academy of Management Annual Meeting* (Washington, DC).

[B63] Dunivin K. O. (1994). Military culture: change and continuity. Armed Forces Soc. 20, 531–547.

[B64] Duval-Lantoine C. Imra-Millei B. (2021). Comprehensive Culture Change in the CAF: From Buzzword to Actionable Items. Canadian Global Affairs Institute. Available online at: https://d3n8a8pro7vhmx.cloudfront.net/cdfai/pages/4693/attachments/original/1620253956/Comprehensive_Culture_Change_in_the_CAF_From_Buzzword_to_Actionable_Items.pdf?1620253956 (accessed August 31, 2023).

[B65] Dyson D. (2023). Canadian Armed Forces facing member shortage 'crisis'. CTV News. Available online at: https://ottawa.ctvnews.ca/canadian-armed-forces-facing-member-shortage-crisis-1.6344761 (accessed March 19, 2024).

[B66] Eagly A. H. (1987). Sex Differences in Social Behavior: A Social-Role Interpretation. New York, NY: mprint Psychology Press. 10.4324/9780203781906

[B67] Eagly A. H. Chaiken S. (1993). The Psychology of Attitudes. San Diego: Harcourt Brace Jovanovich College Publishers.

[B68] Eagly A. H. Wood W. Diekman A. B. (2000). “Social role theory of sex differences and similarities: a current appraisal,” in The Developmental Social Psychology of Gender, eds. T. Eckes & H. M. Traunter (Mahwah, NJ: Lawrence Erlbaum), 123–174.

[B69] Eichler M. (2016). Learning from the Deschamps Report: why military and veteran researchers ought to pay attention to gender. J. Military, Veteran and Family Health. 2, 5–8. 10.3138/jmvfh.3394

[B70] Eisenberg N. Miller P. A. (1987). The relation of empathy to prosocial and related behaviors. Psychol. Bull. 101, 91–119. 3562705

[B71] Eisenberg N. Strayer J. (1987). “Critical issues in the study of empathy,” in Empathy and its Development, eds. N.Eisenberg & J. Strayer (Cambridge: Cambridge University Press), 3–13.

[B72] Elizur D. Guttman L. (1976). The structure of attitudes toward work and technological change within an organization. Administrat. Sci. Quart. 21, 611–623.

[B73] Ely R. J. Thomas D. A. (2020). Getting serious about diversity. Harv. Bus. Rev. 98, 114–122. Available online at: https://hbr.org/2020/11/getting-serious-about-diversity-enough-already-with-the-business-case (accessed March 18, 2024).

[B74] Erwin D. G. Garman A. N. (2010). Resistance to organizational change: linking research and practice. Leadersh. Organ. Dev. J. 31, 39–56. 10.1108/01437731011010371

[B75] Eyre W. (2021). Message from the Acting Chief of the Defense Staff: Update to the Canadian Armed Forces on Culture Change. Available online at: https://www.canada.ca/en/department-national-defence/maple-leaf/defence/2021/07/message-from-acting-cds-update-to-caf-on-culture-change.html (accessed August 31, 2023).

[B76] Eyre W. Matthews B. (2022). Message to Defence Team members: Promoting and Measuring Inclusive Behaviours within the Defence Team. Available online at: https://www.canada.ca/en/department-national-defence/maple-leaf/defence/2022/04/promoting-measuring-inclusive-behaviours.html (accessed August 31, 2023).

[B77] Ferdman B. M. (2017). Paradoxes of inclusion: Understanding and managing the tensions of diversity and multiculturalism. J. Appl. Behav. Sci. 53, 235–263. 10.1177/00218863177026008

[B78] Ferguson R. B. (2021). Masculinity and war. Curr. Anthropol. 62, S108–S120. 10.1086/711622

[B79] Fernandez C. S. Taylor M. M. Dave G. Brandert K. Larkin S. Mollenkopf K.. (2023). Improving the equity landscape at US academic institutions: 10 strategies to lead change. Equity Educ. Soc. 2023, 27526461231215084. 10.1177/27526461231215084 PMC13021095 41907873

[B80] Fors Brandebo M. Börjesson M. Hilmarsson H. (2022). Longitudinal studies on cohesion in a military context–a systematic review. Milit. Psychol. 34, 732–741. 10.1080/08995605.2022.2041995 PMC10013318 38536274

[B81] Gabbiadini A. Riva P. Andrighetto L. Volpato C. Bushman B. J. (2016). Acting like a tough guy: Violent-sexist video games, identification with game characters, masculine beliefs, & empathy for female violence victims. PLoS ONE 11, 1–14. 10.1371/journal.pone.0152121 27074057 PMC4830454

[B82] Gallant J. (2022). “Too white and too male, Canadian Armed Forces are rethinking recruiting as staffing slides, senior officers say,” in Toronto Star. Available online at: https://www.thestar.com/politics/federal/2022/03/23/too-white-and-too-male-canadian-armed-forces-are-rethinking-recruiting-as-staffing-slides-senior-officers-say.html

[B83] Garcia-Barrera M. A. Karr J. E. Trujillo-Orrego N. Trujillo-Orrego S. Pineda D. A. (2017). Evaluating empathy in anadan ex-combatants: examination of the internal structure of the interpersonal reactivity index (IRI) in anada. Psychol. Assess. 29, 116–122. 10.1037/pas000033 27111730

[B84] Gilken J. Johnson H. L. (2019). Supporting belongingness through instructional interventions in community college classrooms. Commun. College Enterprise 25, 32–49. Available online at: https://www.proquest.com/docview/2342433679/abstract/34BFF1FBA4604642PQ/1?accountid=13608&sourcetype=Scholarly%20Journals (accessed March 18, 2024).

[B85] Government of Canada (2022a). About Operation HONOUR, Canada. Available online at: https://www.canada.ca/en/department-national-defence/services/benefits-military/conflict-misconduct/sexual-misconduct/about-operation-honour.html (accessed August 31, 2023).

[B86] Government of Canada (2022b). Canadian Armed Forces Ethos: Trusted to Serve. Available online at: https://www.canada.ca/en/department-national-defence/corporate/reports-publications/anadian-armed-forces-ethos-trusted-to-serve.html (accessed August 31, 2023).

[B87] Government of Canada (2022c). Employment Equity and Diversity in the Department of National Defence and the Canadian Armed Forces Report. Available online at: https://www.canada.ca/en/ombudsman-national-defence-forces/reports-news-statistics/investigative-reports/employment-equity-diversity/employment-equity-diversity-report.html#top (accessed August 31, 2023).

[B88] Government of Canada (2022d). Minister of National Defence Advisory Panel on Systemic Racism and Discrimination – Final Report – January 2022: With a focus on Anti-Indigenous and Anti-Black Racism, LGBTQ2+ *Prejudice, Gender Bias, and White Supremacy*. Available online at: https://www.canada.ca/en/department-national-defence/corporate/reports-publications/mnd-advisory-panel-systemic-racism-discrimination-final-report-jan-2022.html

[B89] Government of Canada (2022e). Statistics of women in the Canadian Armed Forces as of April 2022, Canada. Available online at: https://www.canada.ca/en/department-national-defence/services/women-in-the-forces/statistics.html (accessed August 31, 2023).

[B90] Greco S. von Hlatky S. (2020). “Equity, diversity, and inclusion: revising the concept of military professionalism in the Canadian Armed Forces,” in Rethinking Military Professionalism for the Changing Armed Forces, eds. K. Hachey, T. Libel and W.H. Dean (Eds.) (Cham: Springer), 189–200.

[B91] Gregory T. K. (2022). Is the term “warrior” suitable for the Canadian Armed Forces? Canad. Milit. J. 22, 52–57. Available online at: http://www.journal.forces.gc.ca/PDFs/CMJ224Ep52.pdf (accessed March 18, 2024).

[B92] Hall J. A. Schwartz R. (2019). Empathy present and future. J. Soc. Psychol. 159, 225–243. 10.1080/00224545.2018.1477442 29781776

[B93] Harrison D. Laliberté L. (2002). The First Casualty: Violence Against Women in Canadian Military Communities. Toronto, ON: James Lorimer & Company.

[B94] Hayes A. (2022). Introduction to Mediation, Moderation, and Conditional Process Analysis: A Regression-Based Approach. New York: Guilford Publications.

[B95] Hayes A. (2023). The PROCESS macro for SPSS, SAS, and R. Process Macro. Available online at: https://www.processmacro.org/download.html (accessed August 31, 2023).

[B96] Hays-Thomas R. Bendick M. (2013). Professionalizing diversity and inclusion practice: Should voluntary standards be the chicken or the egg? Indust. Organizat. Psychol. 6, 193–205. 10.1111/iops.12033

[B97] Hinojosa R. (2010). Doing Hegemony: Military, Men, and Constructing a Hegemonic Masculinity. J. Men's Stud. 18, 179–194. 10.3149/jms.1802.179

[B98] Hinton M. Pilkey D. Harpe A. Carter D. Penner R. Ali S.. (2021). Factors that help and factors that prevent Canadian military members' use of mental health services. J. Military, Veter. Family Health 7, 102–109. 10.3138/jmvfh-2020-0055

[B99] Hix W. M. MacCoun R. J. (2010). “Cohesion and performance,” in Sexual Orientation and US Military Personnel Policy: An Update of RAND's 1993 Study, ed. R. Corporatoin (Santa Monica, CA: RAND Corporation), 137–166.

[B100] Jetten J. Branscombe N. R. Spears R. (2002). On being peripheral: effects of identity insecurity on personal and collective self-esteem. Eur. J. Soc. Psychol. 32, 105–123. 10.1002/ejsp.64

[B101] Johansen R. B. Laberg J. C. Martinussen M. (2013). Measuring Military Identity: scale development and psychometric evaluations. Soc. Behav. Personal. 41, 861-880. 10.2224/sbp.2013.41.5.861

[B102] Joiner T. E. Jr. Hollar D. Van Orden K. A. (2006). On Buckeyes, Gators, Super Bowl Sunday, and the Miracle on Ice: “Pulling Together” is associated with lower suicide rates. J. Soc. Clin. Psychol. 25, 180–196. 10.1037/a0016500

[B103] Joseph J. S. Smith-MacDonald L. Filice M. C. Smith M. S. (2023). Reculturation: a new perspective on military-civilian transition stress. Military Psychol. 35, 193–203. 10.1080/08995605.2022.2094175 37133548 PMC10198009

[B104] Joshi A. Son J. Roh H. (2015). When can women close the gap? A meta-analytic test of sex differences in performance and rewards. Acad. Manage. J. 58, 1516–1545. 10.5465/amj.2013.0721

[B105] Judge T. A. Kammeyer-Mueller J. D. (2012). General and specific measures in organizational behavior research: considerations, examples, and recommendations for researchers. J. Organ. Behav. 33, 161–174. 10.1002/job.764

[B106] Kamas L. Preston A. (2021). Empathy, gender, and prosocial behavior. Journal of Behav. Experim. Econ. 92, 101654. 10.1016/j.socec.2020.101654

[B107] Kanter R. M. (1977). Men and Women of the Corporation. New York: Basic Books

[B108] Keats P. A. (2010). Soldiers working internationally: Impacts of masculinity, military culture, and operational stress on cross-cultural adaption. Int. J. Adv. Counsel. 32, 290–303. 10.1007/s10447-010-9107-z

[B109] Kiecolt-Glaser J. K. Ricker D. George J. Messick G. Speicher C. E. Garner W.. (1984). Urinary cortisol levels, cellular immunocompetency, and loneliness in psychiatric inpatients. Psychosom. Med. 46, 15–23. 10.1097/00006842-198401000-00004 6701251

[B110] Koenig L. J. Isaacs A. M. Schwartz J. A. (1994). Sex differences in adolescent depression and loneliness: why are boys lonelier if girls are more depressed?. J. Res. Pers. 28, 27–43. 10.1006/jrpe.1994.1004

[B111] Konrath S. H. O'Brien E. H. Hsing C. (2010). Changes in dispositional empathy in american college students over time: a meta-analysis. Personal. Soc. Psychol. Rev. 15, 180–198. 10.1177/1088868310377395 20688954

[B112] Kovitz M. (2000). The Enemy Within: Female Soldiers in the Canadian Armed Forces. Toronto: Canadian Woman Studies/les cahiers de la femme.

[B113] Lancaster S. L. Kintzle S. Castro C. A. (2018). Validation of the warrior identity scale in the chicagoland veterans study. Identity. 18, 34–43. 10.1080/15283488.2017.1410157

[B114] Lane A. (2017). Special men: The gendered militarization of the Canadian Armed forces. Int. J.l 72, 463–483. 10.1177/0020702017741910

[B115] Leary M. R. Kowalski R. M. Smith L. Phillips S. (2003). Teasing, rejection, and violence: Case studies of the school shootings. Aggres. Behav. 29, 202-214. 10.1002/ab.10061

[B116] Leary M. R. Tambor E. S. Terdal S. K. Downs D. L. (1995). Self-esteem as an interpersonal monitor: the sociometer hypothesis. J. Pers. Soc. Psychol. 68, 518–530.

[B117] Lee S. Y. Hanson M. D. Cheung H. K. (2019). Incorporating bystander intervention into sexual harassment training. Ind. Organ. Psychol. 12, 52–57. 10.1017/iop.2019.810.1017/iop.2019.8

[B118] Lovakov A. Agadullina E. R. (2021). Empirically derived guidelines for effect size interpretation in social psychology. Eur. J. Soc. Psychol. 51, 485–504. 10.1002/ejsp.2752

[B119] Lucas-Molina B. Pérez-Albéniz A. Ortuño-Sierra J. Fonseca-Pedrero E. (2017). Dimensional structure and measurement invariance of the Interpersonal Reactivity Index (IRI) across gender. Psicothema 29, 590–595. 10.7334/psicothema2017.19 29048323

[B120] MacKinnon D. P. Lockwood C. M. Williams J. (2004). Confidence limits for the indirect effect: distribution of the product and resampling methods. Multivariate Behav. Res. 39, 99–128. 10.1207/s15327906mbr3901_4 20157642 PMC2821115

[B121] MacKinnon D. P. Pirlott A. G. (2015). Statistical approaches for enhancing causal interpretation of the M to Y relation in mediation analysis. Personal. Soc. Psychol. Rev. 19, 30–43. 10.1177/1088868314542878 25063043 PMC4835342

[B122] Madsen S. R. Townsend A. Scribner R. T. (2020). Strategies that male allies use to advance women in the workplace. J. Men's Stud. 28, 239–259. 10.1177/1060826519883239

[B123] Mangat R. Momani B. Edgar A. (2020). Unpacking diversity and inclusion. In Strengthening the Canadian Armed Forces Through Diversity and Inclusion. Toronto: University of Toronto Press.

[B124] Maslow A. H. (1968). Toward a Psychology of Being. New York: Van Nostrand.

[B125] Maxwell A. (2020). Experiences of Unwanted Sexualized and Discriminatory Behaviours and Sexual Assault Among Students at Canadian Military Colleges, 2019. Available online at: https://www150.statcan.gc.ca/n1/pub/85-002-x/2020001/article/00011-eng.html (accessed August 31, 2023).

[B126] McCristall P. Baggaley K. (2019). The progressions of a gendered military: A theoretical examination of gender inequality in the Canadian military. J. Military, Veter. Family Health 5, 119–126. 10.3138/jmvfh.2017-0026 PMC13372499 42534069

[B127] McGraw K. (2016). Gender differences among military combatants: Does social support, ostracism, and pain perception influence psychological health?. Mil. Med. 181, 80–85. 10.7205/MILMED-D-15-00254 26741905

[B128] McMaster G. (2023). U of A Professor Recruited to Assess White Supremacy in Canadian Armed Forces. Edmonton, AB: University of Alberta. Available online at: https://www.ualberta.ca/folio/2023/01/u-of-a-professor-recruited-to-assess-white-supremacy-in-canadian-armed-forces.html (accessed August 31, 2023).

[B129] Miller P. A. Eisenberg N. (1988). The relation of empathy to aggressive and externalizing/antisocial behavior. Psychol. Bull. 103, 324–344. 3289071 10.1037/0033-2909.103.3.324

[B130] Milner A. Kavanagh A. King T. Currier D. (2018). The influence of masculine norms and occupational factors on mental health: evidence from the baseline of the Australian longitudinal study on male health. Am. J. Men's Health, 12, 696–705. 10.1177/1557988317752607 29338558 PMC6131428

[B131] Mor Barak M. E. (2011). Managing diversity: Toward a globally inclusive workplace (2nd ed.). Thousand Oaks, CA: Sage.

[B132] Mor Barak M. E. Lizano E. L. Kim A. Duan L. Rhee M. Hsiao H.. (2016). The promise of diversity management for climate of inclusion: a state-of- the-art review and meta-analysis. Human Serv. Organizat. 40, 305–333. 10.1080/23303131.2016.1138915

[B133] Moser C. Branscombe N. R. (2021). Male allies at work: gender-equality supportive men reduce negative underrepresentation effects among women. Soc. Psychol. Personal. Sci. 13, 372–381. 10.1177/F19485506211033748

[B134] Murphy B. A. Costello T. H. Watts A. L. Cheong Y. F. Berg J. M. Lilienfeld S. O. (2020). Strengths and weaknesses of two empathy measures: a comparison of the measurement precision, construct validity, and incremental validity of two multidimensional indices. Assessment 27, 246–260. 10.1177/1073191118777636 29847994

[B135] Murphy M. C. Gopalan M. Carter E. R. Emerson K. T. Bottoms B. L. Walton G. M. (2020). A customized belonging intervention improves retention of socially disadvantaged students at a broad-access university. Sci. Adv. 6, aba467. 32832625 10.1126/sciadv.aba4677 PMC7439303

[B136] Neuberg S. L. Cialdini R. B. Brown S. L. Luce C. Sagarin B. J. Lewis B. P. (1997). Does empathy lead to anything more than superficial helping? Comment on Batson et al. (1997). J. Personal. Soc. Psychol. 73, 510–516.

[B137] Neumann D. L. Chan R. C. Boyle G. J. Wang Y. Westbury H. R. (2015). Measures of empathy: self-report, behavioral, and neuroscientific approaches. Measu. Persona. Soc. Psychol. Constructs 2015, 257–289. 10.1016/B978-0-12-386915-9.00010-3

[B138] Nicol A. A. Rounding K. (2013). Alienation and empathy as mediators of the relation between Social Dominance Orientation, Right-Wing Authoritarianism and expressions of racism and sexism. Personal. Individ. Differen. 55, 294-299. 10.1016/j.paid.2013.03.009

[B139] Nicol A. A. M. Charbonneau D. Boies K. (2007). Right-wing authoritarianism and social dominance orientation in a Canadian military sample. Milit. Psychol. 19, 239-257. 10.1080/08995600701548072

[B140] O'Donohue W. Yeater E. A. Fanetti M. (2003). Rape prevention with college males: The roles of rape myth acceptance, victim empathy, and outcome expectancies. J. Interpersonal Viol. 18, 513-531. 10.1177/0886260503251070

[B141] Office of the Auditor General of Canada (2017). Report 6 — Royal Military College of Canada – National Defence. Available online at: https://www.oag-bvg.gc.ca/internet/English/parl_oag_201711_06_e_42671.html

[B142] Oliver L. W. Harman J. Hoover E. Hayes S. M. Pandhi N. A. (1999). A quantitative integration of the military cohesion literature. Milit. Psychol. 11, 57–83.

[B143] O'Reilly J. Robinson S. L. Berdahl J. L. Banki S. (2015). Is negative attention better than no attention? The comparative effects of ostracism and harassment at work. Organizat. Sci. 26, 774–793. 10.1287/orsc.2014.090

[B144] Pendlebury J. (2020). “This is a man's job”: challenging the masculine “warrior culture” at the U.S. Air Force Academy. Armed Forces & Soc. 46, 163–164. 10.1177/0095327X18806524

[B145] Penner L. A. Dovidio J. F. Piliavin J. A. Schroeder D. A. (2005). Prosocial behavior: Multilevel perspectives. Annu. Rev. Psychol., 56, 365–392. 10.1146/annurev.psych.56.091103.070141 15709940

[B146] Petty R. E. Fabrigar L. R. Wegener D. T. (2003). “Emotional factors in attitudes and persuasion,” in Handbook of Affective Sciences, eds. R. J. Davidson, K. R. Scherer, and H. H. Goldsmith (Oxford: Oxford University Press), 752–772.

[B147] Piderit S. K. (2000). Rethinking resistance and recognizing ambivalence: a multidimensional view of attitudes toward an organizational change. Acad. Manage. Rev. 25, 783–794. 10.5465/amr.2000.3707722

[B148] Pierotti J. (2020). Barriers to women in the Canadian Armed Forces. Canad. Milit. J. 20, 20-31. Available online at: http://www.journal.forces.gc.ca/Vol20/No4/PDF/CMJ204Ep20.pdf (accessed March 18, 2024).

[B149] Pugliese D. (2022a). “Special forces soldier faces scrutiny for alleged support of convoy protesters,” in Ottawa Citizen. Available online at: https://ottawacitizen.com/news/national/defence-watch/special-forces-soldier-faces-scrutiny-for-alleged-support-of-convoy-protesters (accessed August 31, 2023).

[B150] Pugliese D. (2022b). “Canadian Forces officers applaud speech slamming Canada's climate change policies cancel culture, weak leaders,” in Ottawa Citizen. Available online at: https://ottawacitizen.com/news/national/defence-watch/speech-slamming-canadas-climate-change-policies-cancel-culture-and-weak-leaders-applauded-by-canadian-forces-officers (accessed August 31, 2023).

[B151] Rau T. J. Merrill L. L. McWhorter S. K. Stander V. A. Thomsen C. J. Dyslin C. W.. (2010). Evaluation of a sexual assault education/prevention program for male US Navy personnel. Mil. Med. 175, 429–434. 10.7205/MILMED-D-09-00218 20572476

[B152] Rehman M. (2023). Chief of Defence Staff announces 15th CAF Chief Warrant Officer. Canadian Military Family Magazine. Available online at: https://www.cmfmag.ca/policy/chief-of-defence-staff-announces-15th-caf-chief-warrant-officer/ (accessed April 16, 2023).

[B153] Reis J. Menezes S. (2020). Gender inequalities in the military service: a systematic literature review. Sexual. Cult. 24, 1004–1018. 10.1007/s12119-019-09662-y

[B154] Richard K. Molloy S. (2020). An examination of emerging adult military men: Masculinity and U.S. military climate. Psychol. Men Mascul. 21, 686–698. 10.1037/men0000303

[B155] Rosenberg M. J. Hovland C. I. McGuire W. J. Abelson R. P. Brehm J. W. (1960). “Attitude organization and change: an analysis of consistency among attitude components,” in Yales Studies in Attitude and Communication, Vol. III (New Haven: Yale Univer. Press).

[B156] Rosenstein J. E. Angelis K. D. McCone D. R. Carroll M. H. (2018). Sexual assault and sexual harassment at the US Military Service Academies. Milit. Psychol. 30, 206–218. 10.1080/08995605.2017.1422950

[B157] Royal Military College of Canada. (2023). “What is RMC,” in Government of Canada. Available online at: https://www.rmc-cmr.ca/en/college-commandants-office/what-rmc (accessed August 31, 2023).

[B158] Ryan R. M. Deci E. L. (2000). The darker and brighter sides of human existence: Basic psychological needs as a unifying concept. Psychol. Inq. 11, 319–338. 10.1207/S15327965PLI1104_03

[B159] Sadler A. G. Mengeling M. A. Booth B. M. O'Shea A. M. J. Torner J. C. (2017). The relationship between US military officer leadership behaviors and risk of sexual assault of reserve, national guard, and active component servicewomen in nondeployed locations. Am. J. Public Health 107, 147–155. 10.2105/AJPH.2016.303520 27854521 PMC5308164

[B160] Schewe P. A. (2002). “Guidelines for developing rape prevention and risk reduction interventions,” in Preventing violence in relationships: Interventions across the life span, ed. P. A. Schewe (Washington, D.C.: American Psychological Association), 107–136.

[B161] Scoppio G. Otis N. Yan Y. Hogenkamp S. (2022). Experiences of officer cadets in canadian military colleges and civilian universities: a gender perspective. Armed Force Soc. 48, 49–69. 10.1177/0095327X20905121

[B162] Selby E. A. Anestis M. D. Bender T. W. Ribeiro J. D. Nock M. K. Rudd M. D.. (2010). Overcoming the fear of lethal injury: evaluating suicidal behavior in the military through the lens of the Interpersonal-Psychological Theory of Suicide. Clini. Psychol. Rev. 30, 298–307. 10.1016/j.cpr.2009.12.004 20051309 PMC2834834

[B163] Shields D. M. Kuhl D. Westwood M. J. (2017). Abject masculinity and the military: articulating a fulcrum of struggle and change. Psychol. Men Mascul. 18, 215–225. 10.1037/men0000114

[B164] Shore L. M. Randel A. E. Chung B. G. Dean M. A. Holcombe Ehrhart K. Singh G. (2011). Inclusion and diversity in work groups: a review and model for future research. J. Manage. 37, 1262–1289. 10.1177/0149206310385943

[B165] Short N. A. Stentz L. Raines A. M. Boffa J. W. Schmidt N. B. (2019). Intervening on thwarted belongingness and perceived burdensomeness to reduce suicidality among veterans: Subanalyses from a randomized controlled trial. Behav. Ther. 50, 886–897. 10.1016/j.beth.2019.01.004 31422845 PMC6703169

[B166] Singer M. Mitchell S. Turner J. (1998). Consideration of moral intensity in ethicality judgments: Its relationship with whistle-blowing and need-for-cognition. Journal of Business Ethics, 17, 527-541. 10.1023/A:1005765926472

[B167] Siu A. M. Shek D. T. (2005). Validation of the interpersonal reactivity index in a Chinese context. Res. Soc. Work Pract. 15, 118–126. 10.1002/pchj.281

[B168] Statistics Canada (2019). Canadian Armed Forces Regular Force members who were sexually assaulted in the past 12 months, by gender and selected characteristics, 2016 and 2018. Available online at: https://www150.statcan.gc.ca/n1/pub/85-603-x/2019002/tbl/tbl02-eng.htm

[B169] Statistics Canada (2022a). Diversity of Canada's Veterans and military population. Statistics Canada. Available online at: https://www150.statcan.gc.ca/n1/pub/85-002-x/2020001/article/00011-eng.htm# (accessed March 18, 2024).

[B170] Statistics Canada (2022b). Population projections on immigration and diversity for Canada and its regions, 2016 to 2041: Overview of projection assumptions and scenarios. Available online at: https://www150.statcan.gc.ca/n1/pub/17-20-0001/172000012022001-eng.htm

[B171] Stephenson M. Cosset M. A. Connolly A. (2021a). “Former top soldier Gen. Jonathan Vance facing allegations of inappropriate behaviour with female subordinates: sources,” in Global News. Available online at: https://globalnews.ca/news/7614063/anadian-vance-sexual-misconduct-operation-honour/

[B172] Stephenson M. Cossette M. A. Connolly A. (2021b). “Women in military feeling ‘seething undercurrent of rage' over allegations: senior female officer,” in Global News. Available online at: https://globalnews.ca/news/7701880/military-sexual-misconduct-eleanor-taylor-resignation/

[B173] Taber N. (2018). After deschamps: men, masculinities, and the canadian armed forces. J. Military, Veter. Family Health. 4, 100–107. 10.3138/jmvfh.2017-0005

[B174] Taber N. (2022). Trusted to serve: rethinking the CAF ethos for culture change. Canad. Milit. J. 22, 13–19. Available online at: http://www.journal.forces.gc.ca/PDFs/CMJ223Ep13.pdf (accessed March 18, 2024).

[B175] Termium (2023). Diversity. Government of Canada. Available online at: https://www.btb.termiumplus.gc.ca/tpv2alpha/alpha-eng.html?lang=eng&i=1&srchtxt=diversity&index=alt&codom2nd_wet=1#resultrecs (accessed March 18, 2024).

[B176] The Canadian Press (2021). “Gen. Jonathan Vance obstruction of justice case headed for trial in May 2023,” in Global News. Available online at: https://globalnews.ca/news/8335095/anadian-forces-sexual-misconduct-jonathan-vance-trial/

[B177] Thomas D. A. Ely R. J. (1996). Making differences matter. Harv. Bus. Rev. 74, 79–90.

[B178] Twenge J. M. Baumeister R. F. DeWall C. N. Ciarocco N. J. Bartels J. M. (2007). Social exclusion decreases prosocial behavior. J. Personal. Soc. Psychol. 92, 56–66. 10.1037/0022-3514.92.1.56 17201542

[B179] Twenge J. M. Baumeister R. F. Tice D. M. Stucke T. S. (2001). If you can't join them, beat them: effects of social exclusion on aggressive behavior. J. Pers. Soc. Psychol. 81, 1058–1069. 10.1037/0022-3514.81.6.1058 11761307

[B180] Tyler T. R. Blader S. L. (2003). The group engagement model: procedural justice, social identity, and cooperative behavior. Personal. Soc. Psychol. Rev. 7, 349–361. 10.1207/S15327957PSPR0704_07 14633471

[B181] Vachon D. D. Lynam D. R. Johnson J. A. (2014). The (non)relation between empathy and aggression: Surprising results from a meta-analysis. Psychol. Bullet. 140, 751–773. 10.1037/a0035236 24364745

[B182] Van den Broeck A. Sulea C. Vander Elst T. Fischmann G. Iliescu D. De Witte H. (2014). The mediating role of psychological needs in the relation between qualitative job insecurity and counterproductive work behavior. Career Dev. Int. 19, 526–547. 10.1108/CDI-05-2013-0063

[B183] Van Orden K. A. Cukrowicz K. C. Witte T. K. Joiner Jr T. E. (2012). Thwarted belongingness and perceived burdensomeness: construct validity and psychometric properties of the Interpersonal Needs Questionnaire. Psychol. Assessm. 24, 197–215. 10.1037/a0025358 21928908 PMC3377972

[B184] Van Orden K. A. Witte T. K. Gordon K. H. Bender T. W. Joiner T. E. (2008). Suicidal desire and the capability for suicide: tests of the interpersonal–psychological theory of suicidal behavior among adults. J. Consult. Clini. Psychol. 76, 72–83. 10.1037/0022-006X.76.1.72 18229985

[B185] Vance J. (2016). The chief of the defence staff, general jonathan vance, addresses sexual misconduct in the canadian armed forces. Canad. Milit. J. 16, 6–15. Available online at: http://www.journal.forces.gc.ca/vol16/no3/PDF/CMJ163Ep6.pdf (accessed March 18, 2024).

[B186] Waller L. (2021). A Sense of Belonging at Work: A Guide to Improving Well-being and Performance. London: Routledge.

[B187] Walton G. M. Brady S. T. (2020). “The social-belonging intervention,” in Handbook of Wise Interventions: How Social Psychology Can Help People Change, eds. G.M. Walton and A.J. Crum (New York, NY: Guilford Press), 36–62.

[B188] Walton G. M. Cohen G. L. (2007). A question of belonging: race, social fit, and achievement. J. Personal. Social Psychol. 92, 82–96. 10.1037/0022-3514.92.1.82 17201544

[B189] Walton G. M. Cohen G. L. (2011). A brief social-belonging intervention improves academic and health outcomes of minority students. Science, 331, 1447–1451. 10.1126/science.1198364 21415354

[B190] Walton G. M. Murphy M. C. Logel C. Yeager D. S. Goyer J. P. Brady S. T.. (2023). Where and with whom does a brief social-belonging intervention promote progress in college?. Science 380, 499–505. 10.1126/science.ade4420 37141344

[B191] Wang Y. Li Y. Xiao W. Fu Y. Jie J. (2020). Investigation on the rationality of the extant ways of scoring the interpersonal reactivity index based on confirmatory factor analysis. Front. Psychol. 11, 1086. 10.3389/fpsyg.2020.01086 32581942 PMC7284003

[B192] Waruszynski B. T. MacEachern K. H. Raby S. Straver M. Ouellet E. Makadi E. (2019). Women serving in the Canadian Armed Forces: Strengthening military capabilities and operational effectiveness. Canad. Milit. J. 19, 24–33. Available online at: http://www.journal.forces.gc.ca/Vol19/No2/eng/PDF/CMJ192Ep24.pdf (accessed March 18, 2024).

[B193] Wells P. (2021). “A crisis of confidence in the Canadian Armed Forces,” in MacLeans. Available online at: https://macleans.ca/politics/ottawa/a-crisis-of-confidence-in-the-canadian-armed-forces/

[B194] Willer R. Wimer C. Owens L. A. (2015). What drives the gender gap in charitable giving? Lower empathy leads men to give less to poverty relief. Soc. Sci. Res. 52, 83–98. 10.1016/j.ssresearch.2014.12.014 26004450

[B195] Winslow D. Dunn J. (2002). Women in the Canadian Forces: Between legal and social integration. Curr. Sociol. 50, 641–667. 10.1177/0011392102050005003

[B196] Winters M. F. (2014). “From diversity to inclusion: an inclusion equation,” in Diversity at work: The practice of inclusion, eds. B. M. Ferdman and B. R. Deane (Hoboken NJ: Jossey-Bass), 205– 228.

[B197] Wong Y. J. Ho M. R. Wang S. Miller I. S. (2017). Meta-analyses of the relationship between conformity to masculine norms and mental health-related outcomes. J. Counsel. Psychol. 64, 80–93. 10.1037/cou0000176 27869454

[B198] Zhao H. Peng Z. Sheard G. (2013). Workplace ostracism and hospitality employees' counterproductive work behaviors: The joint moderating effects of proactive personality and political skill. Int. J. Hospital. Manage. 33, 219–227. 10.1016/j.ijhm.2012.08.006

